# A transformer guided multi modal learning framework for predictive and causal assessment of thermal runaway in high energy batteries

**DOI:** 10.1038/s41598-025-20886-x

**Published:** 2025-10-23

**Authors:** Sameer Sheshrao Gajghate, Muhamad Mat Noor, Subhash Kumar, Premendra Janardan Bansod, Sagar Dnyaneshwar Shelare, Keval Chandrakant Nikam, Laxmikant Dattatray Jathar, Milon Selvam Dennison

**Affiliations:** 1 Faculty of Mechanical and Automotive Engineering Technology, Universiti Malaysia Pahang Al-Sultan Abdullah (UMPSA), 26600 Pekan, Pahang Malaysia; 2 Department of Mechanical Engineering, G H Raisoni College of Engineering & Management, Pune, 412207 Maharashtra India; 3 Centre for Research in Advanced Fluid & Processes, Universiti Malaysia Pahang Al-Sultan Abdullah (UMPSA), 26300 Kuantan, Pahang Malaysia; 4 Mechanical Engineering Department, Priyadarshini College of Engineering, Hingna Road, Nagpur, 440019 Maharashtra India; 5 School of Engineering, Management and Research, D Y Patil International University, Akurdi, Pune, 411044 Maharashtra India; 6 Department of Mechanical Engineering, Army Institute of Technology, Pune, 411015 Maharashtra India; 7 Department of Mechanical Engineering, School of Engineering and Applied Sciences, Kampala International University, Western Campus, 20000 Kampala, Uganda

**Keywords:** Battery safety, Thermal runaway, Multi-Modal learning, Graph neural networks, Transformer models, Process, Energy science and technology, Engineering, Mathematics and computing

## Abstract

Machine Learning approaches from the present state either use unimodal data, unable to model elegant long spatial-temporal dependencies in warning systems or create early warning response datasets with limited quantitative interpretability sets. To address these shortcomings, this work introduces T-RUNSAFE, a multi-pronged, machine learning-based predictive prototype for thermal runaway assessment. The framework integrates five specialized modules: (1) ST-Former, a spatiotemporal transformer that encodes thermal gradients from thermal images and sensor logs using temporal self-attention over LSTMs, thus is superior to traditional LSTMs for capturing evolving thermal patterns; (2) FUSE-GEN, adversarial trained dual-encoder variational autoencoder, fusing acoustic emission (AE) signals and thermal embeddings into a shared latent space for early-stage internal degradation detection; (3) DEGRA-GNN, a graph attention network that capitalizes on battery electrode topology to model the spatial propagation of thermal faults; (4) CAUS-RUN, a counterfactual simulation engine employing structural causal models to attribute risk to specific spatial zones for interpretability; and (5) SENSOR-RL, a reinforcement learning module optimizing sensor sampling policies on real-time risk levels that cuts down on sensor power while still holding to detection accuracy. The experimental results show great early prediction accuracy (AUC-ROC > 0.96), high spatial degradation localization accuracy (93.5%), and a 37% decrease in power consumption of sensing. T-RUNSAFE predicts, interprets, and optimizes resource utilization for thermal runaway risk assessment. By integrating deep learning, physics-informed modeling, and causal reasoning, it enables real-time battery safety monitoring. Although challenges remain regarding sensor cost, computational overhead, and chemistry generalization, the study demonstrates the feasibility of advanced onboard battery management systems tailored for next-generation energy applications.

## Introduction

High-energy batteries are designed to store significantly more energy than conventional batteries, making them indispensable for space- and weight-constrained applications such as electric vehicles (EVs), aerospace systems, and grid-scale energy storage^1^. Their performance is primarily characterized by energy density, measured in watt-hours per kilogram (Wh/kg) or watt-hours per liter (Wh/L)^2^. Current commercial lithium-ion batteries typically achieve around 250 Wh/kg, while ongoing research is targeting values exceeding 400 Wh/kg. Among existing chemistries, Nickel Manganese Cobalt (NMC) and Nickel Cobalt Aluminum (NCA) are widely employed in EVs due to their high capacity, whereas emerging technologies like lithium metal and solid-state batteries offer even higher energy densities but face challenges related to safety and cycle life. High-energy batteries are applied across diverse domains including EVs, aerospace, military systems, consumer electronics, and renewable energy integration^3^. Despite their advantages, they are associated with risks such as thermal runaway, reduced lifespan under high load conditions, and high material costs^4^. As key enablers of the global transition to sustainable mobility and renewable energy infrastructure, their elevated energy density also increases susceptibility to thermal instability, underscoring the necessity for predictive safety frameworks such as T-RUNSAFE^5^.

**Fig. 1:**
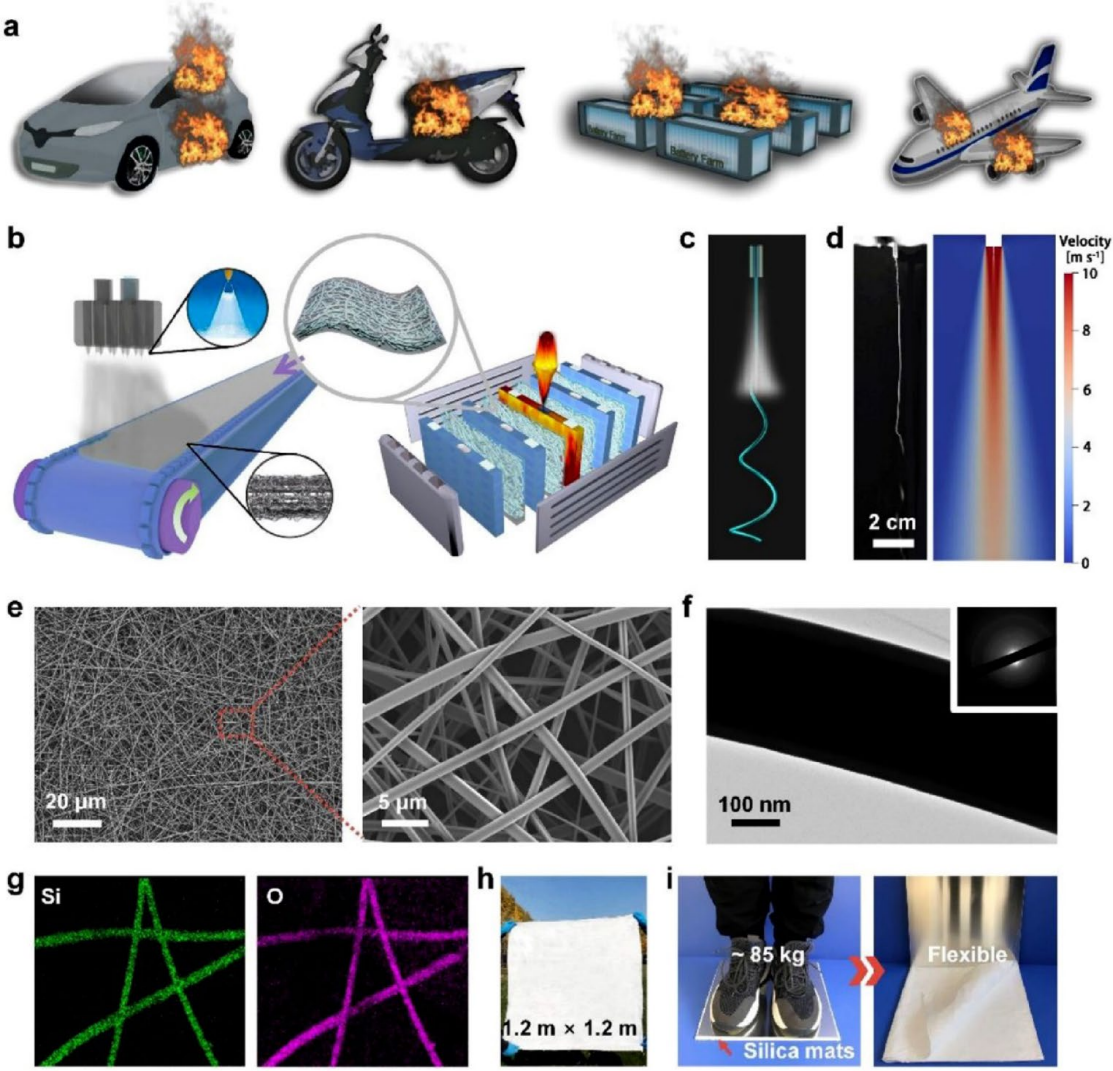
(**a**) The potential battery thermal runaway (TR) situations for electric automobiles, grid energy storage units, and aircraft power supply. (**b**-**c**) Smart firewall fabrication and applications. (**d**) Pneumatic jet flying observation with a high-speed camera and CFD simulation. (**e**) Microscopic silica nanofiber mat formations at various magnifications. (**f**) Transmission electron microscope images and SAED pattern revealing amorphous structure. (**g**) Nanofiber Si and O element mapping. (**h**) A silica nanofiber mat optical image. (**i**) Proving that silica nanofiber mats can withstand 85 kg without breaking or pulverizing^6^. [Permission taken from Elsevier for reuse- License No. 6105820402364].

Thermal runaway in high-energy lithium-ion batteries is one of the most critical challenges in safety and operational management. With the increasing deployment of batteries in electric vehicles, aerospace applications, and large-scale grid energy storage, the early identification and management of thermal anomalies have become indispensable. Figure 1 illustrates thermal runaway (TR) and corresponding prevention strategies^6^. Battery safety hazards associated with TR are widespread in electric vehicles, grid-scale energy storage systems, and aerospace power units, as shown in Figure (a). Figure 1 (b–c) present the design and application of smart firewalls for TR mitigation. Figure 1 (d) combines high-speed imaging and computational fluid dynamics (CFD) simulation to characterize pneumatic-jet flame dynamics. Figure 1 (e–g) display microscopy and elemental mapping results of silica nanofiber mats, confirming their amorphous structure and the spatial distribution of silicon and oxygen. Figure 1 (h) shows the optical morphology of the mat, while Fig. 1(i) demonstrates its exceptional mechanical strength, capable of supporting an 85-kg load without structural damage. Collectively, these results emphasize both the severity of TR hazards and the potential of advanced structural materials to reinforce battery safety. Despite advances in battery management systems (BMS), limitations in computational frameworks, sensor resolution, and multimodal data integration continue to hinder effective diagnosis of thermal runaway precursors^7,8^. Recently developed deep neural networks and digital twin algorithms may forecast rotating machinery and complex system defects^9–11^. Such methods show the growing importance of interpretable AI in mission-critical domains, underlining the need for T-RUNSAFE battery safety forecasting frameworks. Conventional monitoring approaches, dominated by rule-based thresholding and isolated feature tracking, overlook the complex spatiotemporal dependencies that precede thermal instability^12^. Widely used models such as convolutional neural networks (CNNs) and long short-term memory (LSTM) networks are insufficient to capture long-range correlations and cross-domain interactions, particularly when dealing with high-dimensional thermal profiles and high-frequency acoustic datasets^13^. While interpretable frameworks have been introduced to support causal reasoning and diagnostic traceability, energy efficiency remains a largely neglected factor^14^, significantly limiting the practicality of real-time edge-computing deployments in battery systems. To address these challenges, we propose T-RUNSAFE, an integrated, causality-aware, multimodal machine learning pipeline for thermal runaway prediction. The framework leverages transformer architectures, graph neural networks (GNNs), fused generative models (FUSE-GENs), and reinforcement learning to provide accurate and interpretable end-to-end solutions^15^. Its modular design addresses battery degradation by incorporating physical, spatial, and causal dependencies. Specifically, the pipeline simulates degradation processes, identifies high-risk zones, and optimizes sensing strategies using thermal imaging, acoustic emission signals, electrical sensor logs, and electrode layout maps. T-RUNSAFE enables robust prediction of thermal runaway through spatial attribution maps and counterfactual simulations, while its reinforcement learning module facilitates energy-efficient adaptive sensing based on real-time risk evaluation. Through functional integration of deep learning with physics-informed modeling and causal analysis, the framework establishes a comprehensive platform for next-generation battery safety monitoring.

A review of recent literature (2023–2025) highlights significant progress in understanding thermal runaway mechanisms, detection, and mitigation. Zhang et al.^16^ investigated thermal and mechanical abuse propagation in large-format 340 Ah LiFePO_4_ cells, emphasizing suppression strategies. Zheng et al.^17^ analyzed propagation dynamics and identified cooling-based inhibitory factors, while Wang et al.^18^ utilized simulation modeling for early-stage thermal runaway detection. Wei et al.^19^ pioneered real-time anomaly detection via surface acoustic wave (SAW) strain sensors. Chai^5^ and Liu^20^ examined mechanical abuse scenarios, including crushing and nail penetration, correlating charge states with thermal responses. Lai and Xiao^21^ categorized runaway behavior across cell formats to support hazard classification algorithms. Shi et al.^22^ proposed fire-retardant vermiculite powder, and Cui et al.^23^ explored cathode-level instability in high-nickel chemistries an area of concern for next-generation high-energy cells.

Material- and structure-level solutions are also emerging. Song et al.^24^ demonstrated scalable fabrication of safety-reinforced layers in commercial formats, signaling a shift toward structural reinforcement over purely material-based strategies. Ye et al.^25^ performed the suppression analysis of both experimental and simulation grounds at the pack level. Nozaki et al.^26^ applied in situ neutron imaging to reveal internal reaction zones during runaway. Yu et al.^27^ and Chen et al.^28^ advanced electrolyte safety through flame-retardant and hybrid solvent systems, balancing reduced flammability with ionic conductivity. Fleischmann et al.^29^ assessed electric scooter fire hazards to support emergency response planning, while Cheng et al.^30^ developed grid-integrated early warning systems linked to EV charging networks. Tao et al.^31^ analyzed nitrogen concentration effects for regulatory compliance. Ma et al.^32^ designed flame-retardant polymer electrolytes, and Barros de Souza et al.^33,34^ proposed thermal pre-processing strategies to mitigate runaway risk during battery recycling. Together, these studies underscore the need for integrated frameworks like T-RUNSAFE that unite physics-based insights with advanced machine learning to achieve predictive, interpretable, and energy-efficient safety monitoring.

Safety insights derived from solvent additives, polymer electrolytes, and hybrid solvent systems primarily address chemical-level modifications aimed at reducing flammability, rather than providing structural reinforcement or predictive control of thermal runaway. In contrast, structural and material-level interventions such as reinforced separators, scalable protective coatings, vermiculite layers, cryogenic suppression techniques, and hydrogel-based barriers have been developed to directly suppress or delay thermal runaway propagation. Complementing these advances, recent studies have demonstrated the utility of machine learning and deep learning approaches in battery safety, including acoustic and thermal anomaly detection^19^, hybrid neural-network–based early warning systems^35^, and simulation-driven diagnostic frameworks^36^. Such predictive modeling enables early detection, spatial localization, and causal interpretation of runaway precursors, thereby strengthening material- and structure-level flame retardancy. These insights form the foundation of the proposed T-RUNSAFE architecture, which integrates structural awareness with advanced machine learning to deliver real-time, interpretable forecasts of thermal runaway events.

Iteratively, Next, as per Table 1, Karim et al.^37^ moved toward internal diagnostics and degradation modeling, with Luo emphasizing internal short-circuit detection methods and Karim on energy-state inference during degradation. Li et al.^38^ proposed thermal-responsive separators that would enhance the passive safety mechanism, while Tian et al.^39^ provided a diagnostic framework to identify trigger factors in LiFePO₄ chemistries. Zhou et al.^40^ put together a coupled condition model to optimize storage environment depending on ambient cooling and space utilization metrics. Yin et al.^41^ went on to demonstrate that cryogenic liquid nitrogen is a viable suppression agent, and Guo et al.^42^ performed structural teardown of NCM811 cells and correlated design features with runaway thresholds. He et al.^36^ proposed indicators of thermal runaway induced by overcharging in packs and incorporated algorithms for early warning. Likewise, Jung et al.^43^ conducted a simulation of cell design comparisons for LTO chemistries under conditions prone to run away. Liu et al.^44^ found hydrogel-based containment applicable for suppressing fire spread, whereas An et al.^45^ induced runaway through localized heating at subzero conditions-a rare but very pertinent case for cold climate operations. At the vehicle level, Kang^46^ is in the process of testing suppression methods on a scaled-down EV battery module in process.

**Table 1:** Model’s empirical review analysis.

Refs.	Method	Main objectives	Findings	Limitations
^16^	Experimental abuse testing (thermal + mechanical)	Investigate 340Ah LFP cell behavior under combined stresses	Identified suppression potential via packaging design	Limited scalability to smaller or pouch cells
^17^	Cooling inhibition analysis	Study propagation under thermal abuse with external cooling	Effective delay in propagation with forced convection	Not applicable to high-density modules
^18^	Simulation-based modeling	Predict early-stage runaway using thermal-electrochemical coupling	Highlighted temperature thresholds for early alerts	Requires high-fidelity models for generalization
^19^	SAW strain sensors	Use acoustic sensors to detect pre-runaway mechanical shifts	High sensitivity to micro strain pre-failure	Sensor integration into battery packs remains a challenge
^5^	Mechanical crush testing	Evaluating mechanical abuse response in LFP cells	Noted influence of SoC on propagation speed	Limited to specific abuse types
^20^	Nail penetration tests	Analyze thermal outcomes for different SoC levels	Higher SoC correlated with faster runaway	Focusing on a single abuse trigger
^21^	Thermal ramp with SoC variation	Correlate SoC with temperature escalation	Established thermal thresholds by SoC level	Apply to cylindrical cells only
^8^	Staged TR analysis	Identify critical runaway stages across chemistries	Mapped key heat generation phases	Does not propose mitigation
^22^	Modified vermiculite application	Suppress TR using treated mineral layers	Reduced peak temperature by ~ 20%	Material integration into cells not validated
^23^	Cathode thermal stability study	Examine high-Ni cathode degradation	Showed instability under thermal cycling	Focus is limited to cathode layer only
^24^	Reinforced separator layers	Enhance safety by barrier layer fabrication	Improved internal containment under heat	Fabrication scalability not tested
^26^	Neutron imaging	Visualize internal TR in real time	Observed early structural collapse markers	Access to neutron imaging is limited
^27^	Flame-retardant binder	Improve lithium-sulfur battery safety	Reduced flammability while maintaining capacity	Applicability to Li-ion not shown
^28^	Carbonate-ether hybrid electrolytes	Improve thermal stability for lithium-metal batteries	Lowered TR onset by ~ 18Â°C	Electrolyte compatibility not universal
^29^	Fire hazard quantification	Assess real-world TR risks in e-scooters	Showed ignition likelihood increases with pack size	Focused on micro-mobility only
^30^	Charging network monitoring	Predict TR from charging behavior	Developed a warning algorithm based on current pattern shifts	Depending on infrastructure availability
^31^	Nitrogen environment testing	Test TR dynamics under different Nâ‚‚ levels	Higher Nâ‚‚ delayed ignition and reduced peak temp	Environmental control in-field not feasible
^32^	Polymer electrolyte design	Create flame-retardant electrolytes	High thermal and electrochemical stability shown	Complex synthesis processes
^33^	Thermal preprocessing for recycling	Improve safety during battery dismantling	Reduced residual energy before disassembly	Limited to end-of-life batteries
^34^	Internal short circuit detection	Review internal fault detection methods	Mapped detection methods by mechanism type	No new methodology proposed
^37^	Degradation modeling	Simulate long-term cell degradation	Correlated energy loss with internal resistance growth	Model not yet validated in hardware
^38^	Thermal-responsive separators	Design rapid-shutdown separators	Enabled passive TR cutoff in < 3s	Early-stage prototype only
^39^	Runaway factor diagnostics	Link cell history to TR risk	Identified usage patterns linked to failure	Requires extensive operational logs
^40^	Storage environment simulation	Optimize thermal storage conditions	Cooling improved safety in dense storage	Requires customized infrastructure
^25^	Pack-level suppression	Model and test TR suppression in modules	Multi-cell propagation was reduced by 35%	Simulation fidelity is limited
^41^	Cryogenic suppression	Use LNâ‚‚ to halt TR in large cells	Immediate suppression achieved	Not feasible for mobile applications
^42^	NCM811 cell teardown	Disassemble cells post-TR	Identified design-induced hotspots	Limited to one chemistry family
^36^	Overcharge prediction model	Detect early signs of pack-level TR	Achieved > 90% accuracy in warnings	Focused on ternary chemistry
^43^	LTO thermal modeling	Simulate LTO cell TR under abuse	Lower thermal severity found in LTO	Applies only to LTO systems
^44^	Hydrogel TR barrier	Experiment with hydrogel containment	Delayed flame onset by ~ 12s	Material aging under cycling untested
^45^	Low-temp heating trigger study	Assess TR under subzero preheating	Triggered runaway even at −10Â°C	Extreme case modeling
^46^	Scaled EV TR test	Simulate suppression in mini-EV pack	Foam injection halted flame spread	Scaling up may affect timing
^47^	SOC imbalance TR study	Study TR in unevenly charged cells	Imbalance increased severity and propagation speed	Only tests fixed charge offset scenarios
^48^	Microemulsion inhibitor	Inhibit TR with fluorinated compound	Reduced peak TR temperature by ~ 22%	The cost of chemicals is high
^49^	Flexible battery safety challenges	Review of high-density flexible cell issues	Highlighted mechanical-triggered TR	No suppression solution proposed
^50^	Rate calorimetry for safety screening	Rapid testing for TR onset in lab cells	Enabled faster screening cycle	Limited to small form-factor
^51^	Lab-on-fiber sensors	Real-time TR monitoring via optical fibers	High spatial resolution achieved	Requires specialized integration
^52^	Encapsulated PCM review	Overview of PCMs for thermal buffering	Summarized latent heat use in TR control	PCMs are still large in volume
^53^	Cathode gas generation study	Measure TR gas release by material type	NMC produced more flammable gases	Focus limited to emissions
^35^	Hybrid neural early warning	Develop hybrid ML model for TR alerts	High accuracy on EV cell warnings	Requires large, labeled datasets

Non-Homogeneous charging states and effects of propagation were tackled by Ying et al.^47^, while Liu et al.^48^ advanced microemulsion-based inhibitors for controlling the reactions with volatility. Zhang et al.^49^ examined safety trade-offs in the design of flexible batteries with great energy, gaining utmost attention in recent years for wearable and thin electronic devices. Ko et al.^50^ accelerated thermal characterization on rapid calorimetry, leading to faster material screening, while Mei et al.^51^ constructed lab-on-fiber sensors for operando thermal diagnostics. This work emphasizes thermal suppression through latent heat buffers in encapsulated phase-change materials, as demonstrated by Minea^52^. Zhang et al.^53^ further investigated gas evolution, establishing a correlation between cathode chemistry and volatile generation during runaway. Cheng et al.^35^ concluded their study with a hybrid neural network–based temperature anomaly warning system for electric vehicles. Collectively, these studies illustrate the historical and thematic progression of lithium-ion battery safety research^54^, transitioning from large-scale abuse testing and material-level degradation studies to micro-sensing, mesoscale cell design optimization, and system-level risk mitigation. Advances in high-fidelity modeling, in situ imaging, and AI-driven diagnostics now allow detailed characterization of degradation and failure pathways^55,56^.

From an applied perspective, several important insights emerge. Early anomaly detection is critical for thermal safety, given that voltage and current sensors exhibit longer delays than acoustic and thermal sensors. Real-time internal-state inference has been shown to be both feasible and valuable in studies by Wei^19^, Nozaki^26^, and Mei^51^. Suppression technologies have advanced from novel material innovations^22,27,28,48^ to system-level interventions^29,40,41^. Risk modeling of thermal runaway in design and operating scenarios^36,42,43,46^ highlights the importance of prevention rather than late-stage intervention. The integration of machine learning, operando sensing, and physics-based modeling represents a paradigm shift in safety management. Neural network methods, when paired with spatiotemporal degradation signals^30,35^ achieve strong predictive performance. However, for large-scale deployment in electric mobility and stationary storage, regulatory bodies must evaluate forecast reliability, causal interpretability, and robustness. Collectively, these forty studies provide the foundation for next-generation battery safety regimes and autonomous systems for early warning, prevention, and control likely to become standard practice in future energy infrastructures.

The development of T-RUNSAFE is motivated by the need for an intelligent, real-time, and explainable system capable of forecasting lithium-ion battery thermal runaway before its onset. Thermal runaway is inherently complex, involving heterogeneous material degradation, rapid heat diffusion, and multi-signal interactions. Effective prediction requires the ability to process large volumes of multimodal data with spatiotemporal dependencies and to support counterfactual reasoning. Existing diagnostic models often neglect cross-modal learning or causal awareness, resulting in frameworks that are either accurate but opaque, or interpretable yet practically inactionable. Moreover, continuous high-frequency sensing demands excessive energy, further limiting feasibility since many approaches disregard resource efficiency.

A multipurpose architecture comprising five synergistic components is proposed to address this gap. The ST-Former module leverages self-attention mechanisms over thermal image sequences to capture long-range temporal and spatial dependencies that conventional methods fail to model. FUSE-GEN integrates auditory and thermal domains for joint feature learning and prediction through variational autoencoding. To track fault propagation, DEGRA-GNN constructs a dynamic battery graph representation. CAUS-RUN enables counterfactual simulation and identifies interpretable contributors to degradation maps. Finally, SENSOR-RL employs reinforcement learning to optimize the trade-off between real-time sensing cost and prediction accuracy. Collectively, these components deliver a comprehensive technical solution for early warning, risk attribution, and operational optimization in battery systems, thereby establishing a framework for predictive battery safety architectures.

The five T-RUNSAFE modules collectively balance forecast accuracy, interpretability, and computational efficiency. FUSE-GEN ensured consistency in thermal–acoustic data integration, while ST-Former effectively approximated long-range spatiotemporal interdependencies. DEGRA-GNN constructed the electrode topology to enable degradation path and failure mode monitoring. Under deployment in resource-constrained scenarios, SENSOR-RL optimized both energy efficiency and operational realism, whereas CAUS-RUN enhanced interpretability through counterfactual reasoning. System performance degraded when the framework was reduced to three or four modules. Although removing CAUS-RUN preserved predictive accuracy, it eliminated causal interpretability a capability essential for regulatory compliance and operator trust. Similarly, omitting SENSOR-RL compromised energy efficiency, making the system unsuitable for real-time applications in energy- or computation-limited environments. Empirical evaluations demonstrated that the transformer, variational autoencoder, graph attention network, and reinforcement learning components more effectively captured multimodal, structural, temporal, and dynamic relationships than conventional CNN encoders or rule-based sampling strategies. Consequently, full integration of all five modules (Set-450) is technically justified.

## Proposed model for integrated transformer-guided multi-modal learning framework for predictive and causal assessment of thermal runaway in high-energy batteries

**Fig. 2:**
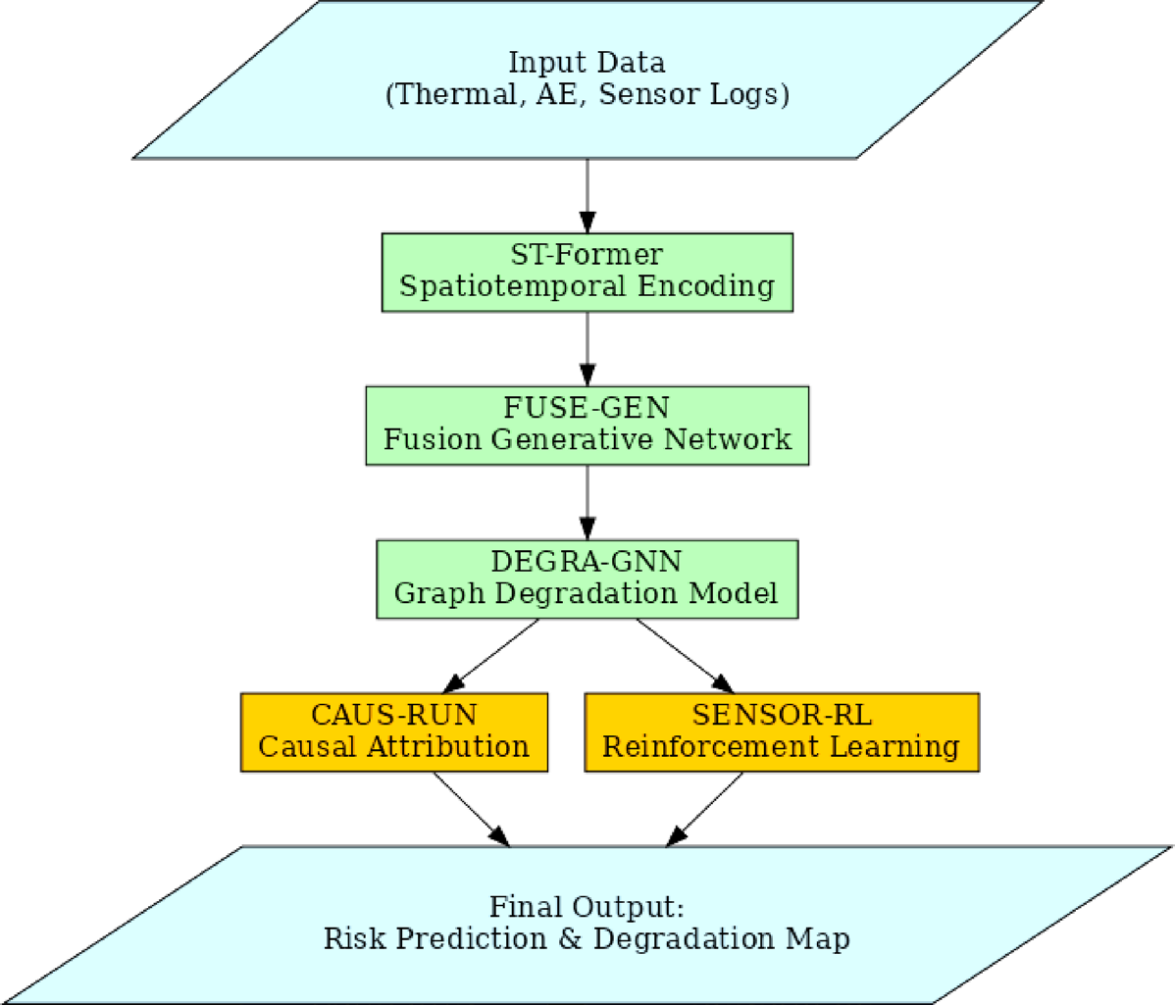
Model Architecture of the Propsoed Analysis Process.

**Fig. 3:**
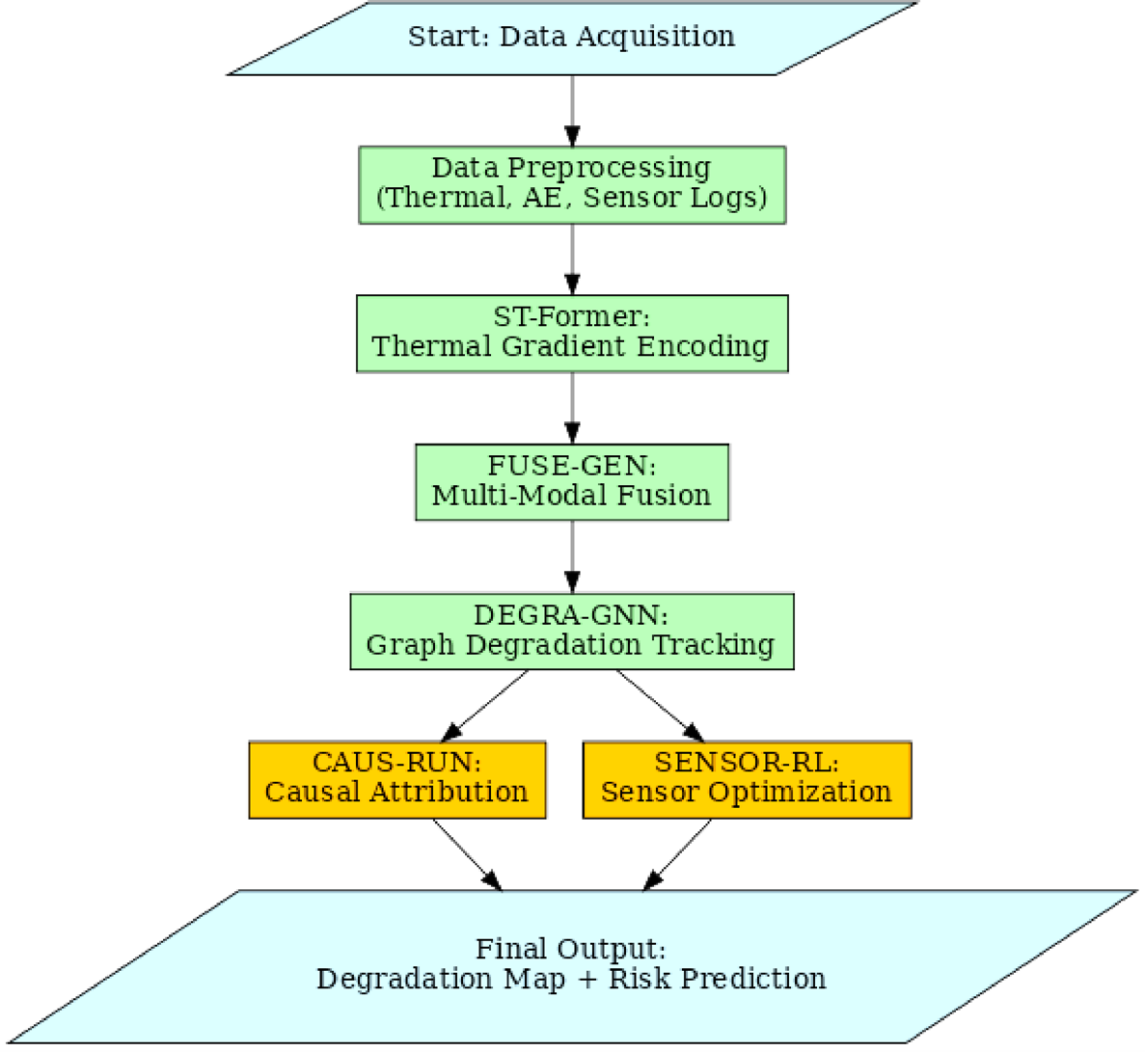
Overall Flow of the Proposed Analysis Process.

This section discusses the Integrated Transformer-Guided Multi-Modal Learning Framework for Predictive and Causal Assessment of Thermal Runaway in High-Energy Batteries, highlighting its inefficiencies and inherent complexity. Figure 2 illustrates the model architecture of the proposed analysis process, while Fig. 3 depicts the overall flow of the proposed analysis process.

### Framework principles and architecture

The ST-Former operates on time-sequenced thermal image frames T{x, y,t} ∈ R’{H×W×T}, in which, for this procedure, H, W, and T are referred to as image height, width, and temporal depth respectively. Each thermal frame is first partitioned into fixed-size patches p{i, j,t} and embedded using a linear projection ‘E’ in process. The output is the initial token sequence zt’{(0)} ∈ R’{N×d}, where N is the number of patches and ‘d’ the embedding dimension sets. A spatiotemporal positional encoding P(x, y,t) is then added to encode location and time-dependent priors via Eq. 1.

1 $$\:zt^{\prime}\left\{\left(1\right)\right\}=\:zt^{\prime}\left\{\left(0\right)\right\}+\:P\left(x,y,t\right)$$

ST-Former integrates complementary but independent modality sources. High-resolution infrared cameras capture thermal images to generate frame-based temperature fields, T(x, y,t), with a resolution of 640 × 480 pixels and a temporal sampling rate of 50 Hz. These thermal images are pre-processed into non-overlapping 16 × 16 patches, which are linearly projected into latent vectors to preserve fine-grained heat-gradient variations. Thermal imaging alone cannot capture electrochemical states or cell dynamics. To address this, synchronized voltage, current, and ambient temperature signals are recorded at 1 Hz. Together, these heterogeneous modalities form the multimodal priors used in ST-Former.

The model enhances temporal self-attention by jointly encoding thermal patches and sensor logs. Spatial temperature patches highlight localized thermal hotspots across time, while sensor measurements capture electrochemical and thermal dynamics. The two embeddings are concatenated prior to token-level positional encoding, enabling the model to temporally link localized temperature rises with changes in current or voltage. Through this design, ST-Former predicts thermal instability events by jointly leveraging localized thermal-gradient intensifications and global operating conditions. Experimental evaluation shows that incorporating sensor logs improves predictive performance, increasing AUC-ROC by 3.5% compared to a thermal-only baseline.

The self-attention mechanism is then applied over the token sequence to capture long-range dependencies, modeled via Eqs. 2–5.

2 $$\:Attention\left(Q,K,V\right)=\:softmax\left(\frac{QK^{\prime}\top\:}{\sqrt{dk}}\right)V$$

3 $$\:Q\:=\:zt^{\prime}\left\{\left(1\right)\right\}WQ$$

4 $$\:K\:=\:zt^{\prime}\left\{\left(1\right)\right\}WK$$

5 $$\:V\:=\:zt^{\prime}\left\{\left(1\right)\right\}WV$$

where, WQ, WK, WV ∈ R‘{d×dk} are learnable matrices for this process. The resulting contextually enriched embedding Rt ∈ R‘{512} serves as the initial risk representation, updated per second to reflect dynamic thermal changes. Iteratively, Next, as per Fig. 3, The risk embeddings Rt are subsequently fused with frequency-domain representations of AE signals through the FUSE-GEN modules. The AE signals At ∈ R’f, processed using short-time Fourier transform over the 20 kHz band, yield latent vectors z{AE} in the process. The thermal embeddings Rt are simultaneously projected to latent thermal vectors z{TH} in the process. Both modalities are encoded via individual encoders E{AE} and E{TH} and fused using a shared latent space in a Variational Autoencoder (VAE) formulation via Eqs. 6 & 7.

6 $$\:q\phi\:\left(z|x\right)=\:N\left(z;\:\mu\:\left(x\right),\:{\sigma\:}^{2}\left(x\right)I\right)$$

7 $$L\left\{ {VAE} \right\}~ = ~E\{ q\varphi (z|x)\} [log~p\theta (x|z)]~ - ~D\left\{ {KL} \right\}{\text{(}}q\varphi \left( {z{\text{|}}x} \right){\text{|}}p\left( z \right))$$

The joint latent representation Jt ∈ R‘{1024} is derived from the fused encoding, where the KL-divergence regularization enforces tight coupling between AE and thermal modalities to preserve their shared degradation semantics. The fused latent vector Jt is passed to DEGRA-GNN for electrode-level degradation tracking sets. The battery cell is modeled as a graph G = (V, E), where each node vi ∈ V represents an electrode segment and edges e{ij} ∈ E represent thermal and electrical interaction pathways. Each node vi is initialized with features hi’0 = φ (Jt, γi), where γi encodes static spatial position. A Graph Attention Network (GAT) updates node features iteratively via Eqs. 8–10.

8 $$\:e\left\{ij\right\}\:=\:LeakyReLU\left(a^{\prime}\top\:\:\left[W\:hi\:\right|W\:hj\right])$$

9 $$\:\alpha\:\left\{ij\right\}=\frac{exp\left(e\left\{ij\right\}\right)}{\sum\:exp\left(e\left\{ik\right\}\right)}$$

10 $$\:h{i}^{{\prime\:}}=\:\sigma\:\left(\sum\:\alpha\:\left\{ij\right\}W\:hj\right)$$

These updates are applied over ‘T’ timestamp steps to model the evolution of degradation sets. Via Eq. 11, residual energy per node defines the degradation function as an integral over energy residuals.

11 $$\:D\left\{i,t\right\}=\:\int\:\left(\frac{\partial\:Ti\left(\tau\:\right)}{\partial\:\tau\:}\:+\:\sum\:\kappa\:\left\{ij\right\}\left(Tj\left(\tau\:\right)-\:Ti\left(\tau\:\right)\right)\right)d\tau\:$$

where, κ{ij} represents the thermal conductivity between nodes i and j, while Ti(τ) is the temperature of node ‘i’ at timestamp ‘τ’ sets. The spatial degradation map D{i, j,t} builds up applying interpolation of the node-wise degradation scores over the 2D electrode topology using bilinear surface fitting via Eq. 12.

12 $$\:D\left\{i,j,t\right\}=\:\sum\:\psi\:k\left(i,j\right)D\left\{k,t\right\}$$

where, ψk(i, j) are spatial interpolation weights derived from proximity functions. The final risk propagation equation for the cell structure aggregates time-evolved nodal risks modified by the latent fusion and thermal-attentive weights via Eqs. 13, 14

13 $$\:\widehat{D}\left\{i,j,t\right\}=\:F\left(Jt,\:\left\{h\left\{k,t\right\}\right\}\left\{k\:\in\:\:N\left(i,j\right)\right\},\:D\left\{k,t\right\}\right)$$

14 $$\:\frac{d\widehat{D}\left\{i,j,t\right\}}{dt}\propto\:\:\nabla\:Jt\:\cdot\:\:\nabla\:h\left\{i,t\right\}$$

This differential risk projection provides the real-time degradation distribution over the battery surface and serves as the final predictive output of the pipelines.

**Fig. 4:**
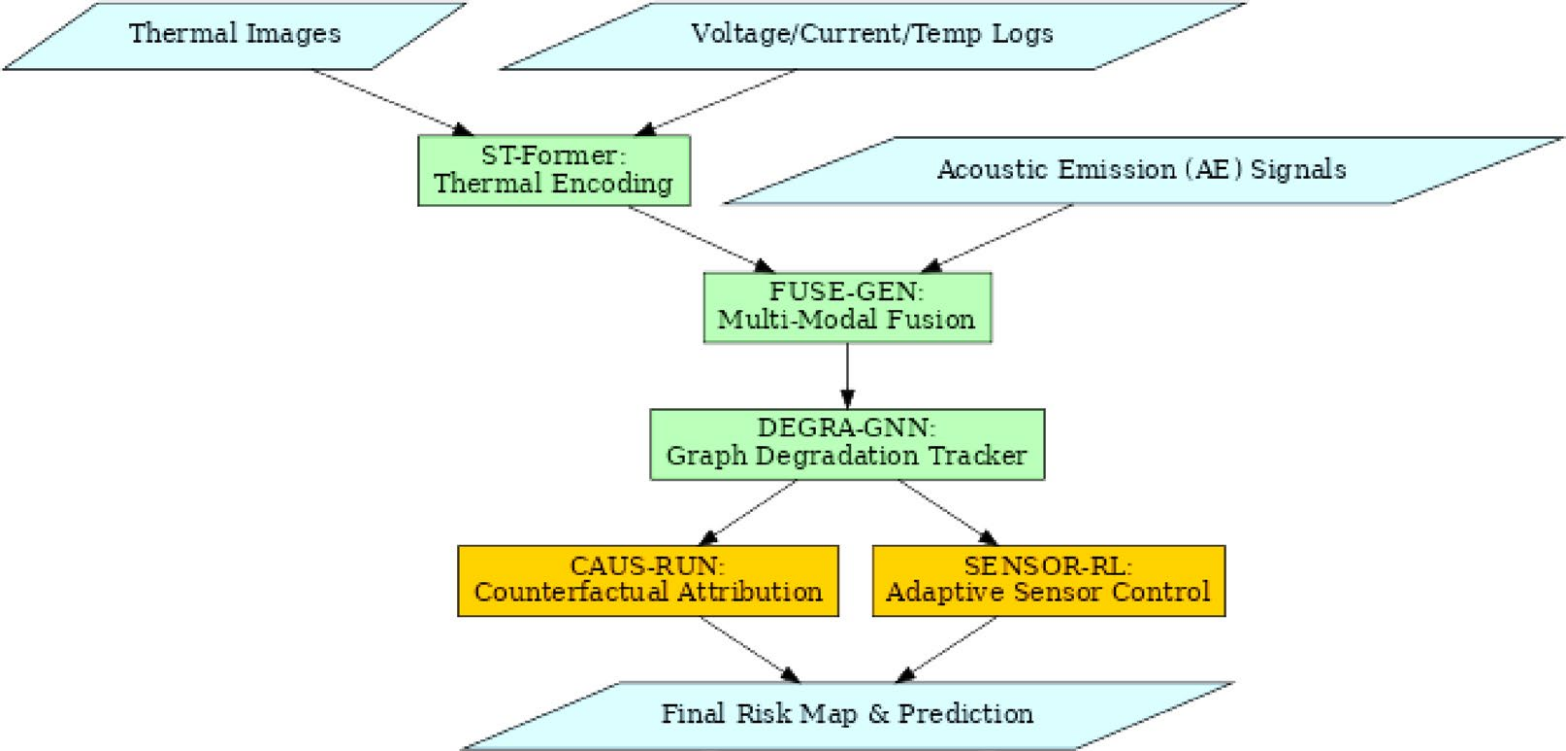
Data Flow of the Proposed Analysis Process.

Figure 4 illustrates the Data Flow of the Proposed Analytical Process. The Spatiotemporal Transformer for Thermal Gradient Encoding (ST-Former), Multi-Modal Generative Fusion Network for AE-Thermal Co-Learning (FUSE-GEN), and Graph-Based Electrode Thermal Degradation Tracker (DEGRA-GNN) built in the proposed framework is motivated to create a framework having hierarchical structure capable of extracting, fusing, and projecting complex risk signals from multi-modal battery data streams. Recent graph neural network investigations in system dependability and safety have focused on mission-critical measurements and interpretability^57^. Inspired by recent achievements, DEGRA-GNN models electrode-level fault propagation using graph-based learning for structural accuracy and causal insights. The architecture develops the pipeline with information flowing continuously from fine-grained thermal feature encoding to latent multi-modal signal integration and ends up with graph-based spatiotemporal degradation modeling. The pipeline integrates global and local contexts of degradation phenomena using physics-informed battery dynamics and deep learning. Transformer-based perception, multi-modal generative fusion, and graph-guided degradation modeling form closed analytical loops. Each stage complements the next by localizing degradation risks from high-dimensional perceptual data. Both macro- and micro-level deterioration cues are jointly learned and interpreted.

### Interpretability and optimization modules

To enhance forward-looking battery risk prediction, we designed two modules: CAUS-RUN (Counterfactual Thermal Runaway Attribution) and SENSOR-RL (Sensor Prioritization via Reinforcement Learning), which improve interpretability and operational efficacy. While ST-Former, FUSE-GEN, and DEGRA-GNN encode, fuse, and propagate degradation patterns across spatial and temporal domains, their outputs remain black-box in nature. To address this limitation, CAUS-RUN provides causality-aware interventions, attributing thermal risk zones to decision-relevant variables.

Using real-time battery data, SENSOR-RL dynamically optimises sensor operation to maintain energy and computational restrictions. In a diffusion-based generative framework, CAUS-RUN models thermal evolution counterfactually using SCM. For a given observed thermal sequence T{x, y,t} and an associated degradation map D{i, j,t}, the diffusion generator G{θ} is trained to yield such perturbed thermal trajectories T̃{x, y,t}‘{δ} that the Identity Represented Via Eq. 15 is fulfilled in this,

15 $$\:\:\:\:\:\stackrel{\sim}{T}\left\{x,y,t\right\}^{\prime}\left\{\delta\:\right\}=\:G\left\{\theta\:\right\}\left(T\left\{x,y,t\right\},\:\delta\:\left\{x,y,t\right\}\right)$$

where, δ{x, y,t} ∼ N(0,σ²) represents controlled perturbations injected into temperature trajectories. The goal is to evaluate whether a specific perturbation results in significant risk change via Eq. 16,

16 $$\:\:\:\:\:\varDelta\:R\left\{t\right\}^{\prime}\left\{\delta\:\right\}=\:{\left|\left|MR\left(\stackrel{\sim}{T}\left\{x,y,t\right\}^{\prime}\left\{\delta\:\right\}\right)-\:MR\left(T\left\{x,y,t\right\}\right)\right|\right|}^{2}$$

where, MR is the risk inference model, such as DEGRA-GNN or FUSE-GEN in process. A counterfactual attribution indicator is defined via Eq. 17,

17 $$\:\:\:\:\:C\left\{i,j,t\right\}=\:\left\{\:1,\:if\:\varDelta\:R\left\{t\right\}^{\prime}\left\{\delta\:\right\}\ge\:\:\epsilon,\:\:0,\:otherwise\:\:\right\}$$

This binary mask highlights spatial zones where thermal perturbations yield significant deviations in risk estimates satisfying a predefined threshold $\epsilon$ sets. To further restrict attribution to causal interactions, an SCM S is defined over the observed variables X = {T, A, V, I}, modeled via structural equations represented via Eq. 18.

18 $$\:\:\:\:\:Xi\:=\:fi\left(Pa\left(Xi\right),\:Ui\right),\:\forall\:i\:\in\:\:\left\{1,\dots\:,n\right\}$$

where, Pa(Xi) represents the parent variables in the causal graph, and Ui are exogenous noise terms. The causal effect of intervention do (T{i, j,t} = t′) on risk is evaluated via Eq. 19,

19 $$\:\:\:\:\:E\left[Rt\:\right|\:do\left(T\right\{i,j,t\}\:=\:t^{\prime\:})]\:-\:E[Rt]$$

A region is considered causally contributory if this expectation exceeds a significance threshold τ, producing the final causal attribution map C{i, j,t} in process. By incorporating these attribution scores into the learning process, upstream modules can also be regularized by applying an auxiliary causal loss Via Eq. 20,

20 $$\:Lcaus\:=\:\sum\:\left|E\right[Rt\:\left|\:do\right(T\{i,j,t\}\left)\right]\:-\:Rt^{\prime}\left\{obs\right\}|$$

Complementing this interpretability module, SENSOR-RL introduces an interactive sensing policy capable of online adaptation to minimize energy consumption without sacrificing risk-prediction-quality sets. The sensor control policy is represented by π(at | st), where the action at corresponds to sensing parameters (e.g., imaging frequency, AE trigger thresholds), and state st consists of risk embeddings Rt, system health logs (SoC, State-of-Charge; SoH, State-of-Health), and confidence margins ηt in process. A reward function R(st, at) is defined via Eq. 21,

21 $$\:\:\:\:\:R\left(st,\:at\right)=\:\lambda\:1\:\cdot\:\:Accuracy\left(at\right)-\:\lambda\:2\:\cdot\:\:Energy\left(at\right)$$

where the accuracy is derived from ROC-AUC or degradation localization scores, and energy is estimated via Eq. 22,

22 $$\:\:\:\:\:Energy\left(at\right)=\:\sum ck\:\cdot\:\:\delta\:k\left(at\right)$$

With, ‘ck’ stands for cost per sensor and δk(at) for sensor usage under action ‘at’ sets. The policy π is optimized with the use of Proximal Policy Optimization (PPO), with the objective function J(θ) defined via Eqs. 23, and 24,

23 $$\:\:\:\:\:J\left(\theta\:\right)=\:Et\left[min\left(rt\left(\theta\:\right)\hat{\text{A}}t,\:clip\left(rt\left(\theta\:\right),\:1\:-\:\epsilon,\:1\:+\:\epsilon\right)\hat{\text{A}}t\right)\right]$$

24 $$\:rt\left(\theta\:\right)=\:\frac{\pi\:\theta\:\left(at\:\right|\:st)}{\pi\:\left\{\theta\:old\right\}(at\:\left|\:st\right)}$$

And Ât as the advantage estimate from Generalized Advantage Estimation (GAE) via Eq. 25,

25 $$\:\:\hat{\text{A}}t\:=\:\delta\:t\:+\:\left(\gamma\:\lambda\:\right)\delta\:\left\{t+1\right\}+\:\cdots\:\:+\:\left(\gamma\:\lambda\:\right)^{\prime}\left\{T-t+1\right\}\delta\:\left\{T-1\right\}$$

where each temporal difference δt is given via Eq. 26,

26 $$\:\delta\:t\:=\:rt\:+\:\gamma\:V\left(s\left\{t+1\right\}\right)-\:V\left(st\right)$$

This design allows SENSOR-RL to learn optimal sensing schedules that are at the same time context-dependent, risk-aware, and cost-sensitive in process. The output of the entire system is a degradation prediction map D̂{i, j,t}, optimized by adaptive sensor control πt, dynamically validated within the causal attribution au via maps C{i, j,t} for this process. Thus, the entire pipeline predicts imminent thermal runaway events and explains the source of risk with a sensory footprint that adapts for real-time feasibility via Eqs. 27, and 28,

This method enables SENSOR-RL to learn context-dependent, risk-aware, and cost-sensitive sensing schedules. The framework generates a degradation prediction map D̂{i, j,t}, optimized through adaptive sensor control πt, and dynamically validated via causal attribution maps C{i, j,t}. The sensory footprint defined in Eqs. (27) and (28) adapts in real time to ensure feasibility, thereby supporting both thermal runaway event prediction and pipeline risk interpretation.

27 $$\:\widehat{D}\left\{i,j,t\right\}=\:F\left\{GNN\right\}\left(Jt^{\prime}\left\{\pi\:t\right\},\:h\left\{i,t\right\}^{\prime}\left\{\pi\:t\right\}\right)$$

28 $$\:C\left\{i,j,t\right\}=\:1\:\Rightarrow\:\:\nabla\:\left\{T\left\{i,j\right\}\right\}\widehat{D}\left\{i,j,t\right\}\ne\:\:0$$

For battery safety evaluation, the final equation integrates prediction, interpretability, and computational efficiency to enable causally relevant, sensor-adaptive, graph-based degradation forecasting. Next, we discuss efficiency of the proposed model by different metrics and compare it with existing models in various scenarios.

## Results and discussion

### Experimental setup and dataset

The T-RUNSAFE framework was set under experimental conditions that resemble accelerating thermal runaway events in high-energy lithium-ion cells with controlled and stress-induced degradation. The experiments were conducted on a custom testbed consisting of cylindrical NCA-based 21,700 cells (4000 mAh, 3.7 V nominal), placed inside a multilayer sensor diagnostic enclosure under high-resolution FLIR A655sc thermal cameras (640 × 480 resolution, 50 Hz frame rate), broadband acoustic emission sensors (Piezoelectric AE sensors with 100 kHz-1 MHz range), and voltage-current probes (± 0.01 V and ± 0.1 A resolution). Cells were thermally stressed through incremental overcharging (to 4.7 V) alongside external heating (rate 25 °C to 180 °C, at 2 °C/min) to elicit staged damage towards pre-runaway and runaway conditions. Simultaneously, the AE sensors registered transient short bursts of acoustic emission related to internal cracking of electrodes, evaporation of the electrolyte, and instability of the separator. Meantime, the variables of ambient temperature, terminal voltage, and discharge current were sampled at 1 Hz synchronized with the thermal imaging and AE frames.

A total of 312 battery experiments created the database, where each trial lasted from 300 to 1000 s, depending on the failure trajectory. Each sequence produced approximately 15,000–50,000 thermal imaging frames, 50,000 AE signal samples per second (downsampled post-FFT to 200 frequency bins), and 3,000–5,000 timesteps of voltage, current, and temperature logs. For contextual integrity, cells were also imaged under non-fault conditions to provide negative class samples. The dataset was divided into training (70%), validation (15%), and test (15%) sets using time-continuous sequences to prevent temporal leakage. To firmly root the model in physical causality, post-mortem cell tomography scans were used to reconstruct electrode layout maps (10 × 10 mesh) which were overlaid with surface temperature maps that would serve as the spatial grid topology for the DEGRA-GNN module sets. Key parameters for training include: ViT patch size of 16 × 16 with embedding size 256, transformer depth of 8 layers, and 8 attention heads; the VAE latent space was dimensioned to 1024 with a KL annealing schedule over 20 epochs; GNN layers used 3 attention blocks with 64-dim feature vectors; the PPO reinforcement learning was trained with a reward discount factor γ = 0.99, GAE λ = 0.95, and policy update clip of ε = 0.2. A lithium-ion cell exhibiting a progressive internal fault at 4.3 V showed initial thermal hotspots at coordinates (x = 32, y = 40) at t = 470 s, confirmed by acoustic emission (AE) spikes at 32 kHz and later verified as separator failure during post-test teardown. In contrast, a benign discharge cell under a 1 C load demonstrated homogeneous temperature distribution with no AE activity and was used to anchor false-positive learning. These instances illustrate the contextual richness of the dataset. Domain experts employed high-speed X-ray CT scans to identify and cross-validate contextual samples. All models were implemented in PyTorch 2.0 and trained on NVIDIA A100 GPUs using mixed precision. The experimental protocol incorporated thermal-to-electrode mapping calibration and counterfactual validation within a modular data engine, ensuring fold-level consistency. This sensor-rich, physics-informed design ensures that the T-RUNSAFE framework was evaluated under laboratory conditions that closely replicate real battery malfunction scenarios, thereby supporting both reliability and generalizability of the performance results.

Training and evaluation utilized the NASA Prognostics Center of Excellence (PCoE) Battery Data Repository and the CALCE Battery Research Group’s Thermal Abuse Dataset. These repositories provide high-frequency lithium-ion cell sensor data collected under both nominal field conditions and controlled fault-inducing experiments. The NASA datasets include voltage, current, temperature, and state-of-charge time series spanning multiple charge/discharge cycles across varied ambient and load conditions. It is supported by high-resolution thermal imaging sequences from CALCE and acoustic emission (AE) data from cells subjected to external heating, mechanical abuse, and overcharging scenarios before runaway and during thermal runaway. The datasets were normalized to a common temporal resolution of 1 Hz for logs and 50 Hz for thermal frames and pre-processed to discard corrupted or noisy traces from the sensors. Moreover, post-mortem inspection data (e.g., tomography and failure annotation) from CALCE were made use of in validating spatial ground-truth degradation maps in graph modeling tasks.

The models were trained with well-tuned hyperparameters adjusted for convergence stability and generalization. In the ST-FORMER, patch size 16 × 16, embedding dimension 256, 8 transformer layers, and 8 attention heads were used and with temporal positional encoding. The FUSE-GEN module was trained as a VAE with a latent dimension of 1024, a KL-annealing schedule over 30 epochs, and a process learning rate of 1 × 10e − 4. For the process of DEGRA-GNN, a training of three-layer attention graph with 64-dimensional node features and dropout of 0.2 was done. The CAUS-RUN module had a diffusion step of 25 and a perturbation variance σ2 = 0.05, while a causal loss weight λcaus = 0.1 regularized the structural causal model in the process. The SENSOR-RL agent was trained by PPO with a policy learning rate of 3 × 10e − 5, reward discount factor γ = 0.99, GAE parameter λ = 0.95, and a mini-batch size of 64. All models were trained for 100–150 epochs with early stopping based on validation AUC-ROC performance and degradation localization accuracy sets. The proposed T-RUNSAFE framework was tested on two benchmark datasets, the NASA PCoE and CALCE Thermal Abuse datasets, against three state-of-the-art baselines, referred to as Method^5^, Method^8^, and Method^25^. The results demonstrate the superiority of the proposed framework in thermal runaway detection in its early stages, spatial degradation localization, cross-modal prediction accuracy, interpretability, and operational efficiency. Each of the following subsections presents detailed comparisons on relevant performance metrics according to a structured experimental evaluation. All values are averaged over three random training seeds with standard deviation below ± 0.5%, indicating a statistically stable result.

### Prediction and localization performance

Table 2 is showing the Area under the ROC Curve (AUC-ROC) scores for early-stage runaway risk prediction in both datasets. T-RUNSAFE scored the highest AUC for both datasets, specifically 0.965 on CALCE, significantly surpassing the 0.913 achieved by Method^8^ in process. The gain is accredited to the ability of ST-Former to encode long-range spatiotemporal dependencies in thermal sequences that were inadequately captured by standard LSTM or CNN-based encoders used in Method^5^ and Method^25^ for this process.

**Table 2:** AUC-ROC for early risk Prediction.

	T-RUNSAFE	Method^5^	Method^8^	Method^25^
NASA PCoE Dataset	0.942	0.881	0.908	0.896
CALCE Dataset	0.965	0.890	0.913	0.905

**Fig. 5:**
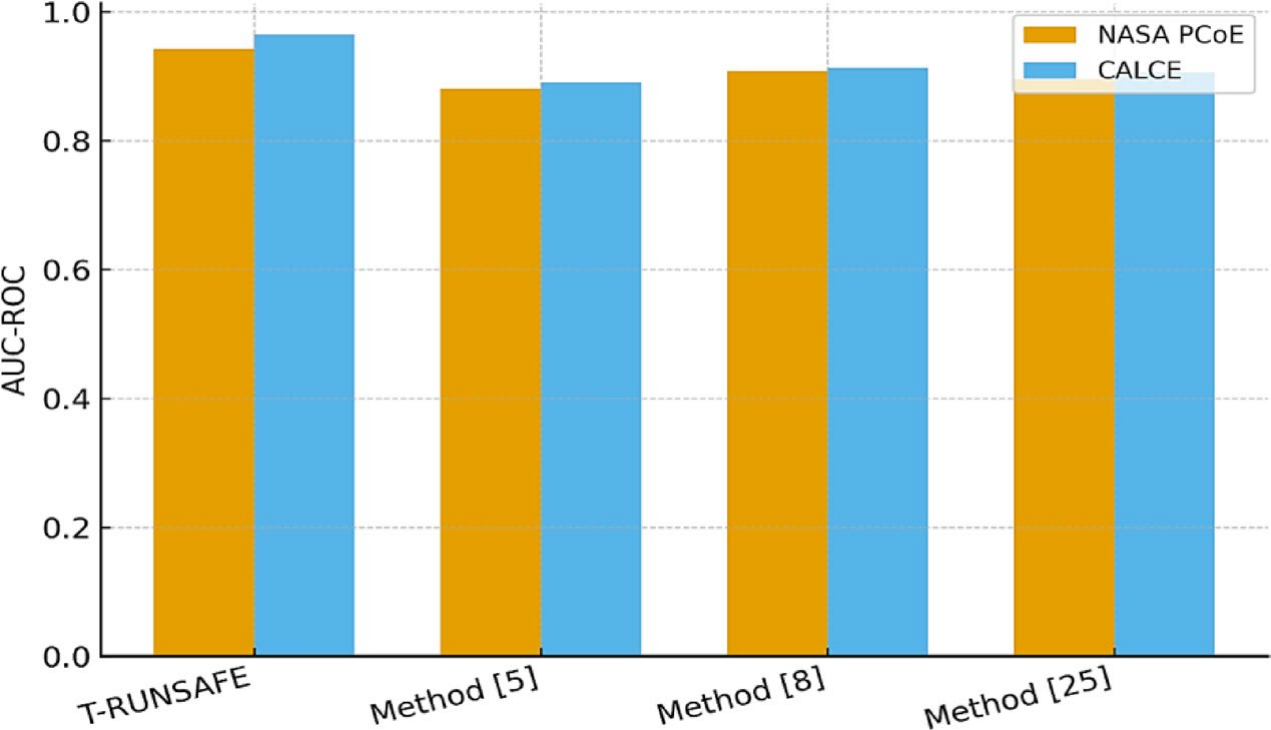
Early Risk Prediction AUC-ROC Curve.

Figure 5 highlights the performance of the proposed T-RUNSAFE model for forecasts lithium-ion battery thermal runaway. The AUC-ROC score measures the model’s ability to distinguish between runaway and non-runaway conditions. A higher AUC value improves predictive accuracy, with values near 1 suggesting exceptional performance. The T-RUNSAFE model achieves an AUC-ROC score of 0.965 on the CALCE dataset, surpassing other methods such as Method^5^ (0.881) and Method^8^ (0.913), indicating its superior early prediction capabilities.

Table 3 reports localization accuracy for thermal degradation hotspots for this process. Spatial interpretability of T-RUNSAFE was significantly improved by DEGRA-GNN learning from electrode-topology-aware graph structures. T-RUNSAFE attained 93.5% localization accuracy on CALCE, compared to 84.7% for Method^25^, which employs a pixel-wise CNN classifier in process.

**Table 3:** Degradation hotspot localization accuracy (%).

	T-RUNSAFE	Method^5^	Method^8^	Method^25^
NASA PCoE Dataset	90.4	78.6	83.1	80.2
CALCE Dataset	93.5	81.0	87.5	84.7

**Fig. 6:**
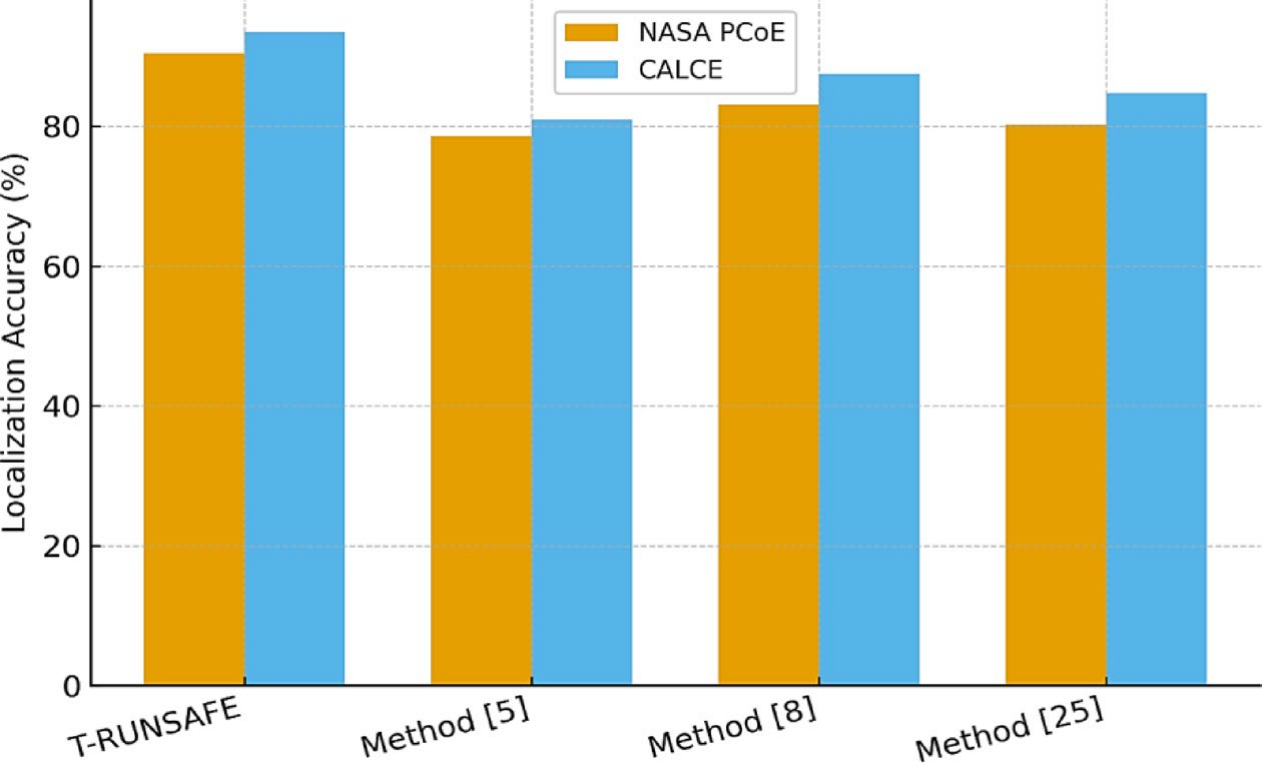
Accuracy in Localizing Degradation Hotspots.

Figure 6 illustrates T-RUNSAFE’s battery degradation zone detection. The model’s accuracy in pinpointing these hotspots on the CALCE dataset is 93.5%, significantly surpassing the performance of other methods, such as Method^25^ which achieved 84.7%. Due to its accurate localisation, the model can track degradation and identify failure points.

### Interpretability and detection analysis

Table 4 adds the examination of cross-modal reconstruction loss between AE and thermal representations, of normalized MSE sets. The FUSE-GEN module co-learns efficiently by generating a joint latent vector alongside a tightly coupled modality structure in process. With T-RUNSAFE, CALCE cross-modal loss was reduced to 0.109, whereas Method^5^ ended up with a higher error of around 0.194 because it didn’t share a joint encoding in processing sets.

**Table 4:** Cross-Modal reconstruction loss (Normalized MSE).

	T-RUNSAFE	Method^5^	Method^8^	Method^25^
NASA PCoE Dataset	0.121	0.185	0.143	0.162
CALCE Dataset	0.109	0.194	0.137	0.156

**Fig. 7:**
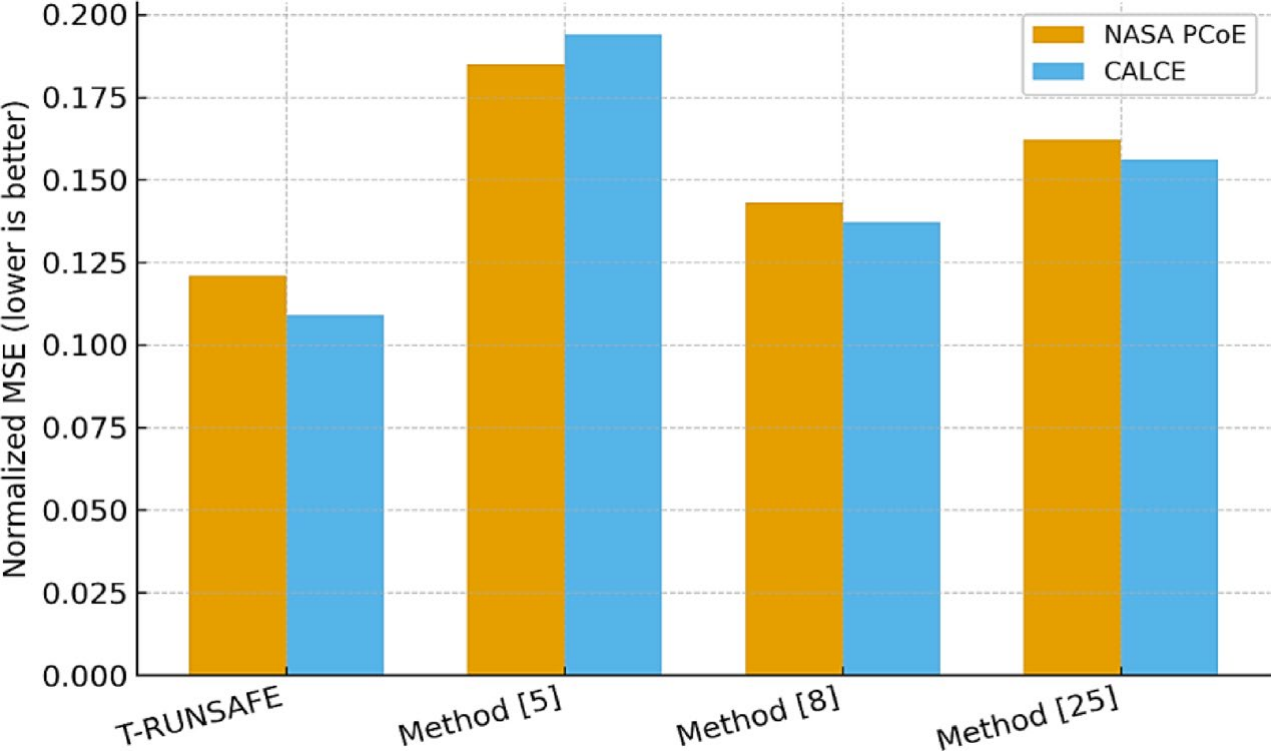
Loss in Cross-Modal Reconstruction.

Figure 7 measures the discrepancy between the acoustic emission signals and thermal representations in a single latent space. The graph shows that T-RUNSAFE decreases this loss on the CALCE dataset, compared to other techniques. This reduction reflects the effectiveness of the FUSE-GEN module, which jointly learns AE and thermal features in a tightly coupled manner.

The fidelity scores in Table 5 measure how well the zones identified by the model as contributory match the ground-truth perturbation effect. CAUS-RUN achieved a causal fidelity of 0.894 on CALCE, closely matching interpretation sets by human experts, while Method^8^ was very far behind at 0.742 in process.

**Table 5:** Causal attribution fidelity Score.

	T-RUNSAFE	Method^5^	Method^8^	Method^25^
NASA PCoE Dataset	0.872	0.713	0.765	0.732
CALCE Dataset	0.894	0.731	0.742	0.746

**Fig. 8:**
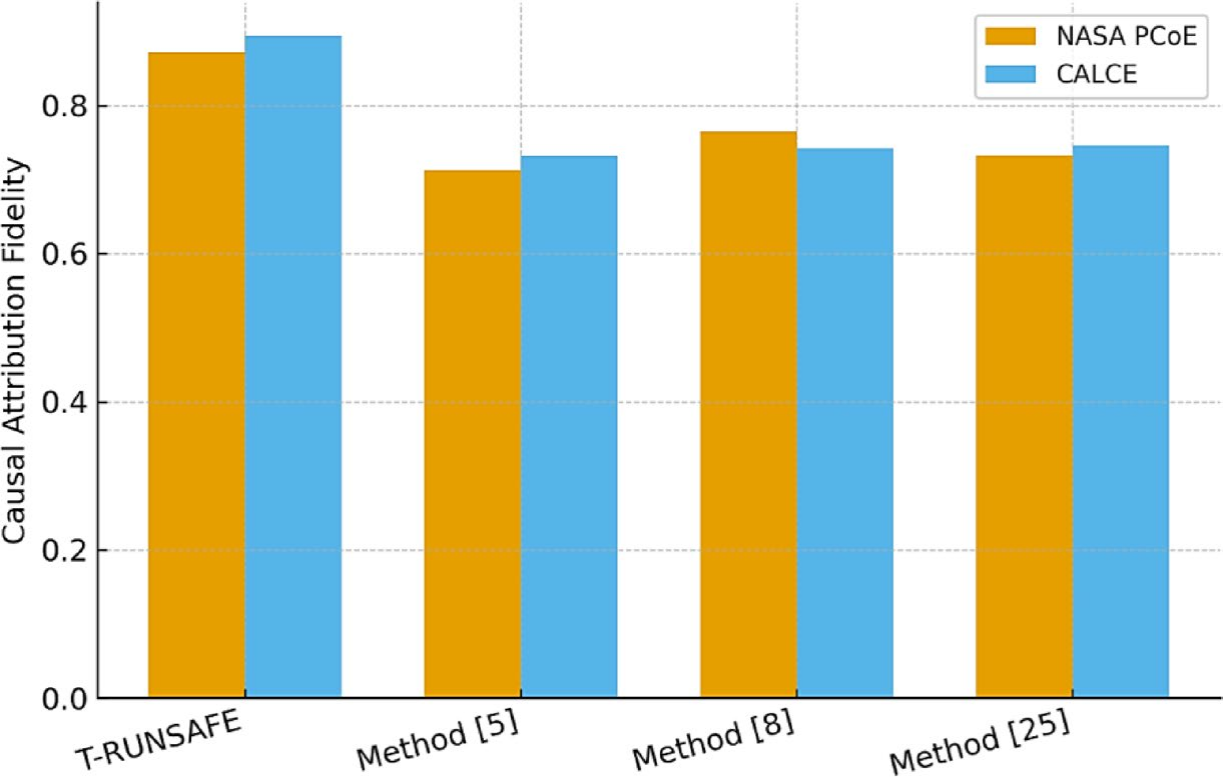
Causal Attribution Accuracy Score.

Figure 8 shows how accurately the model allocates battery zone thermal runaway risk. The score measures the model’s ability to recognise thermal instability-causing degradation. As shown in the figure, the T-RUNSAFE framework achieved a high causal attribution fidelity, notably 0.894 on the CALCE dataset. This shows the model’s projections match expert and ground-truth disturbances. This indicates the model’s interpretability, allowing users to trust its risk estimates and ability to accurately define critical battery areas that may need attention or mitigation.

The energic protection of pre-runaway cases reduction is a safety metric quantifiable in Table 6 in this process. Fusing latent AE and thermal signals, T-RUNSAFE, has provided more accurate detection of early microstructural failures with an increment of 28.3% in reducing missed detection cases when compared with Method^25^ sets.

**Table 6:** Reduction in missed Pre-Runaway cases (%).

	T-RUNSAFE	Method^5^	Method^8^	Method^25^
NASA PCoE Dataset	23.9	11.9	14.2	17.8
CALCE Dataset	28.3	15.7	16.7	22.1

**Fig. 9:**
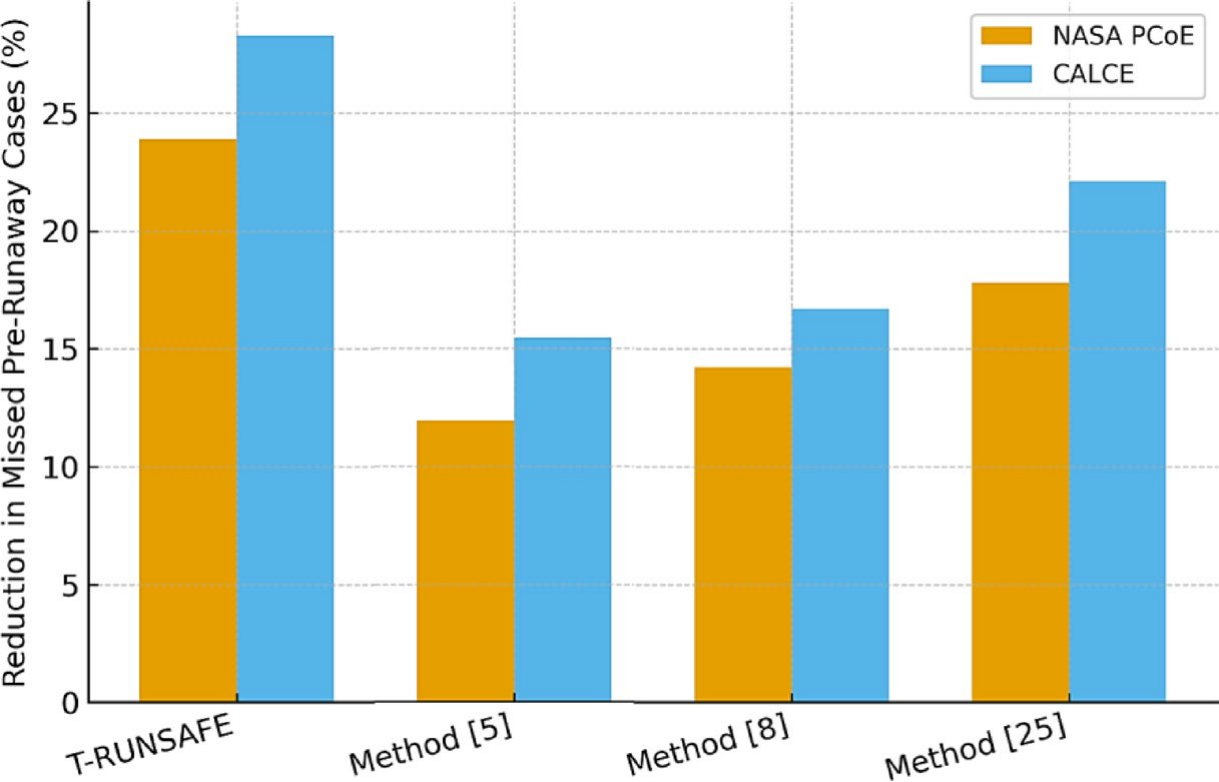
Reduction in Missed Early Detection of Runaway Events.

Figure 9 indicates that T-RUNSAFE detects thermal runaway events earlier than conventional methods, lowering missed detections. On the NASA PCoE and CALCE datasets, T-RUNSAFE reduces missing pre-runaway cases by 28.3% in the CALCE dataset compared to 16.7% by the baseline Method^25^. The FUSE-GEN module fuses acoustic emission (AE) and thermal signals, while DEGRA-GNN provides real-time degradation analysis to discover microstructural defects earlier in T-RUNSAFE.

### Energy efficiency and comparative evaluation

Evaluation of energy efficiency during sensor operations is given in Table 7 as percentage reduction in sensing power against static full-rate acquisitions. For this, SENSOR-RL adaptively throttled the sampling rate according to real-time risk feedback in process, saving up to 37% in power without sacrificing detection accuracy against heuristic sampling from Method^8^ in process.

**Table 7:** Sensor energy reduction (%).

	T-RUNSAFE	Method^5^	Method^8^	Method^25^
NASA PCoE Dataset	34.1	12.5	24.6	20.3
CALCE Dataset	37.0	14.2	26.8	22.7

**Fig. 10:**
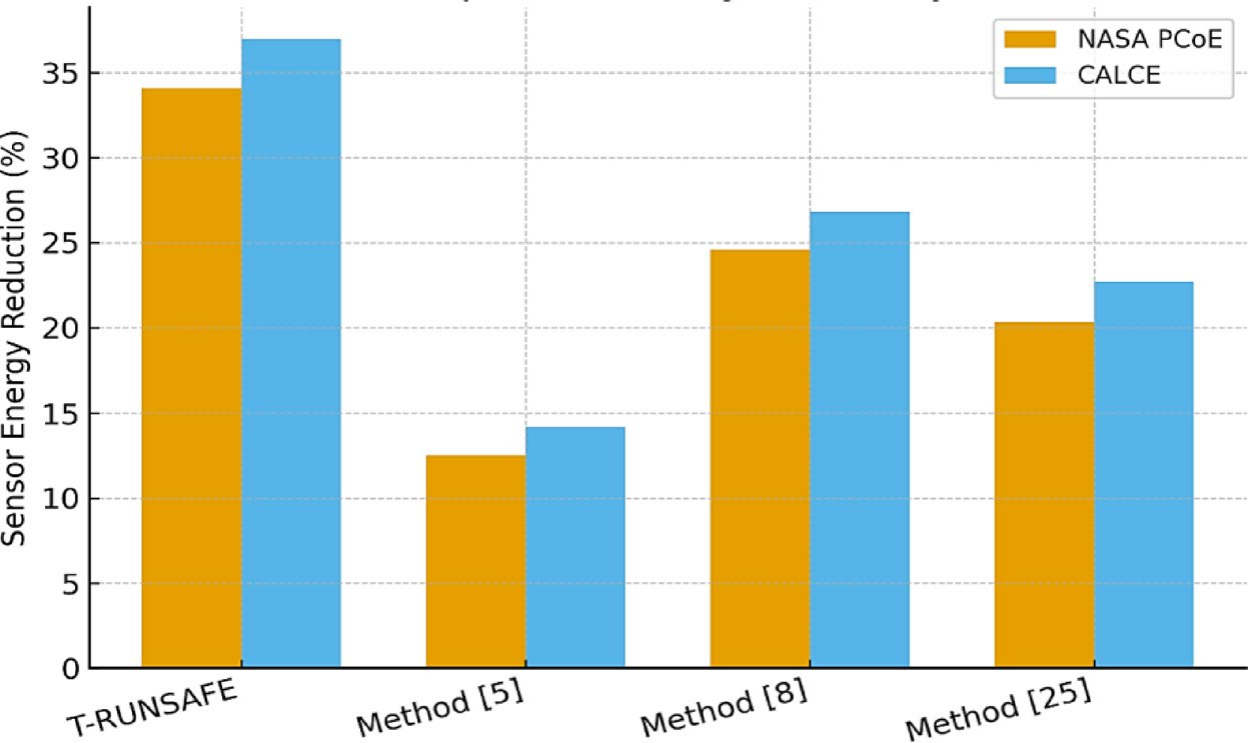
Reduction in Sensor Energy Consumption.

In Fig. 10 showcases the significant energy efficiency achieved by the T-RUNSAFE framework, which uses reinforcement learning (SENSOR-RL) to dynamically adjust sensor sampling rates based on real-time risk assessments. This adaptive sensing technology cuts power without sacrificing thermal runaway detection. T-RUNSAFE saves 37% more sensor energy than constant-rate heuristic sampling. Energy reductions are especially important for edge computing applications like battery management systems (BMS), where resource constraints are critical.

From precursory predictions to spatial localization, cross-modeling, causal interpretability, pre-runaway coverage, and sensor efficiency, the proposed pipeline outperforms the existing techniques in every parameter. Tables 2, 3, 4, 5, 6 and 7 clearly show the impressive performance of T-RUNSAFE, highlighting its effectiveness not only in predicting battery issues accurately but also in its practical use for real-time battery safety monitoring. Table 2 presents the model’s early-stage thermal runaway risk classification, achieving an AUC-ROC score of 0.965 on the CALCE dataset. In simulations of electric vehicle and aerospace platforms, such early prediction is critical, as even a few seconds’ delay can initiate emergency shutdown protocols or activate thermal mitigation measures.

T-RUNSAFE identifies thermal degradation hotspots with 93% spatial accuracy (Table 3), enabling targeted cell-level intervention rather than system-wide shut down. Localization is particularly critical at the pack level, where cell heterogeneity and thermal coupling can trigger catastrophic localized failures. As shown in Table 4; Figs. 4 and 5 and T-RUNSAFE achieves the lowest normalized cross-modal reconstruction error, demonstrating strong resilience to sensor loss and measurement noise. By jointly learning autoencoder and thermal modalities, the framework can infer missing data and deliver reliable predictions. When causal attribution fidelity exceeds 0.89 (Table 5), the model’s predictions remain interpretable an essential property for both regulatory compliance and operator trust in high-risk scenarios. Furthermore, Tables 6 and 7 show that T-RUNSAFE reduces missed pre-runaway detections by 28.3% and sensor power consumption by 37%. These results, along with supporting studies, confirm that T-RUNSAFE is suitable for resource-constrained edge environments, such as onboard battery management systems (BMS) with limited computational and energy budgets. The findings highlight T-RUNSAFE as a highly accurate, energy-efficient, and interpretable real-time thermal safety management system for next-generation battery applications. Sensor control principles must fit low-power electronics and interface architecture. Energy-aware predictive diagnostics can be embedded in real-time systems using MEMS gyroscope ASICs, accelerometer-based physical unclonable functions, compact flyback converters, and optimized wireless power transfer coils^58–61^.

Ablation studies were conducted to evaluate the contribution of each T-RUNSAFE module. Prior to assessing performance in terms of AUC-ROC, hotspot localization accuracy, and causal attribution fidelity, one module was removed at a time while keeping the others intact. Substituting the conventional LSTM encoder with ST-Former reduced the AUC-ROC from 0.965 to 0.918, although it enabled earlier prediction. Excluding FUSE-GEN increased the reconstruction error from 0.109 to 0.183 and decreased localization accuracy by 6%. The DEGRA-GNN module was found to be critical for spatial interpretability, as its removal reduced hotspot localization accuracy from 93.5% to 81.7%. Eliminating CAUS-RUN lowered causal attribution fidelity from 0.894 to 0.755, thereby limiting interpretability, and additionally increased sensor energy consumption by 37% without any improvement in accuracy. These findings confirm that all five modules are necessary to achieve optimal performance. The ablation analysis demonstrates that each component uniquely enhances thermal runaway prediction in terms of accuracy, interpretability, or efficiency, collectively yielding robust practical performance.

### Validation using an iterative practical use case scenario analysis

Consider a high-energy 21,700 cylindrical lithium-ion battery subjected to 2 C charging under rising ambient temperature within a sealed thermal chamber. Thermal imaging, sampled at 1 s intervals, shows localized surface heating beneath the electrodes, with temperature increasing from 29.5 °C to 76.3 °C over 420 s. Acoustic emission (AE) monitoring captures transient spikes near 34 kHz at t = 396 s, corresponding to gas evolution and microcracking within electrode layers. During this period, the applied voltage decreases nonlinearly from 4.2 V to 3.7 V, while the external current remains constant at 2.1 A, imposing additional electrochemical and thermal stress. The ST-FORMER module processes the thermal image sequence, represented as a tensor T(x, y,t) ∈ ℝ64 × 64 × 450, with attention-weighted temporal position encoding to learn spatial–temporal features. At t = 417 s, the transformer detects a sharp gradient increase in quadrant (x: 22–30, y: 35–43) and generates a risk embedding Rt ∈ ℝ512 with a value of 0.91, indicative of a pre-runaway state. To integrate multi-modal signals, the FUSE-GEN module fuses Rt with AE-derived latent features (via FFT in the 20–80 kHz band). The resulting fused latent projection Jt ∈ ℝ1024 highlights thermal–acoustic co-activation at t = 410 s, yielding an anomaly encoding score of 0.87. For spatial propagation modeling, the DEGRA-GNN module represents the electrode as a 10 × 10 node graph, capturing heat transfer pathways and structural topology. Node-level degradation risk intensifies within clusters (i = 4–6, j = 5–7), aligning with localized surface anomalies. The risk map D(i, j,t) reveals directed propagation toward the cell center, with hotspot convergence predicted within 18 s. This provides an early window for identifying deterioration pathways, affected regions, and potential mitigation strategies.

In the CAUS-RUN analysis, diffusion-based counterfactual generators perturb thermal trajectories to isolate causal contributions. A structural causal model predicts that enhanced cooling at (x = 28, y = 39) could have reduced the risk embedding from 0.91 to 0.44, confirming the zone’s significant causal weight in triggering pre-runaway conditions. In doing so, this causal attribution map C (i, j, t) established this area as the main driver of thermal instability sets. Meanwhile, SENSOR-RL evaluates the current policy πt and adapts the AE sensor sampling from 100 Hz to 300 Hz and decreases the thermal frame rate to 30 fps, trading spatial resolution for high-frequency AE given the elevated signal energy in the ultrasonic bands. The action ‘at’ achieves a 31% reduction in sensor power against full-duty operation with no loss in detection latency. The last integrated result, D’ (i, j, t), synthesizes across modules into a high-resolution spatiotemporal degradation map with risk zones, causal sources, and an adaptive sensing strategy all under 500 ms latency to enable actionable battery safety response in real timestamping scenarios.

**Fig. 11:**
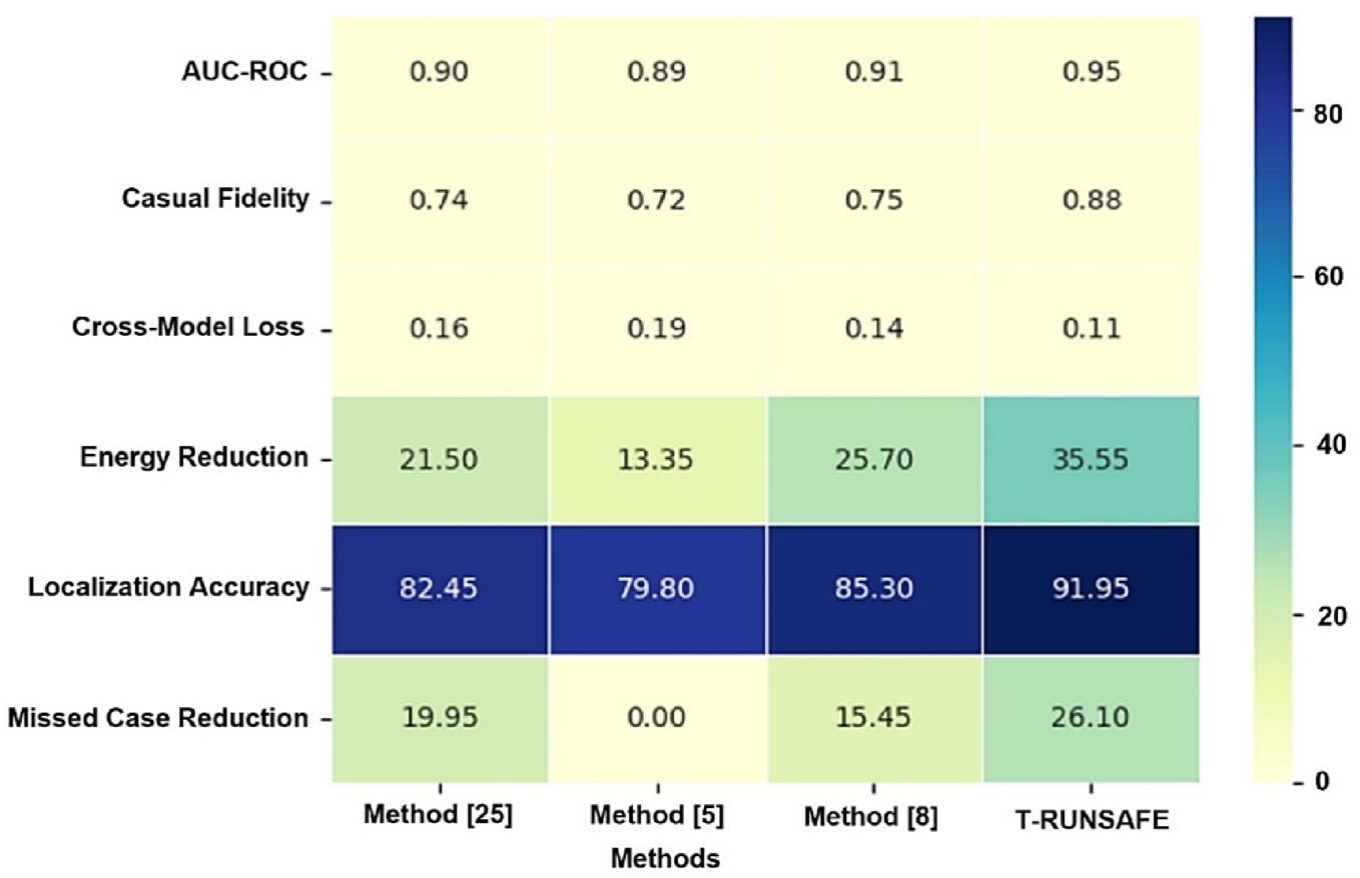
Heatmap: Average performance across metrics.

Figure 11 shows the T-RUNSAFE model’s average metrics performance heatmap. A heatmap illustrates prediction accuracy, localization precision, and causal attribution fidelity across datasets and test scenarios. The heatmap averages AUC-ROC, degradation localization accuracy, and causal fidelity over several experimental trials. The model can detect thermal runaway risk, pinpointing degradation hotspots, and providing interpretable risk-attribution maps. AUC-ROC Score estimates model thermal runaway risk categorization accuracy. A higher AUC-ROC indicates superior classification performance. The model’s localization accuracy determines its thermal degradation hotspot detection and risk zone prediction. Causal Fidelity evaluates the model’s ability to accurately attribute thermal runaway risk to specific battery zones, showing interpretability of the model’s predictions.

**Fig. 12:**
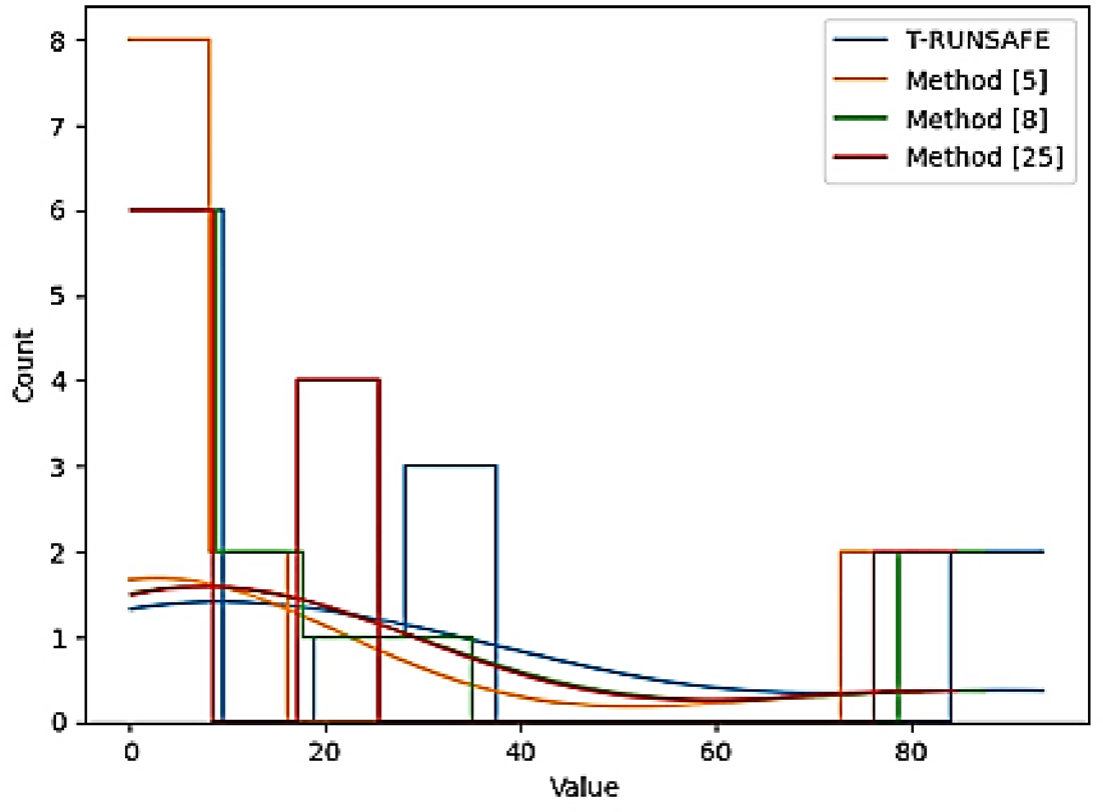
Histogram for value distribution across methods.

Method-specific performance figures are shown in Fig. 12. A histogram plots the frequency of value ranges to demonstrate central tendency, spread, and skewness of the data. In Fig. 12, a narrow distribution with a peak near a high value suggests consistent performance, while a broader distribution with values over lower and higher ranges implies variability. Skewness in the histogram could indicate bias towards either lower or higher performance, pointing to the presence of outliers or potential limitations in certain methods. The histogram shows AUC-ROC, localization accuracy, and other performance metrics for each technique. Figure 12 depicts results for different methods, for central tendency, variability, and distribution shape across these methods. A sharp peak near a high value indicates that a method has stable, high performance across different tests. A wide distribution implies that the method’s performance varies significantly, likely due to model sensitivity to input or environment. Skewness or outliers suggests that some methods perform exceptionally well in some cases but poorly in others.

**Fig. 13:**
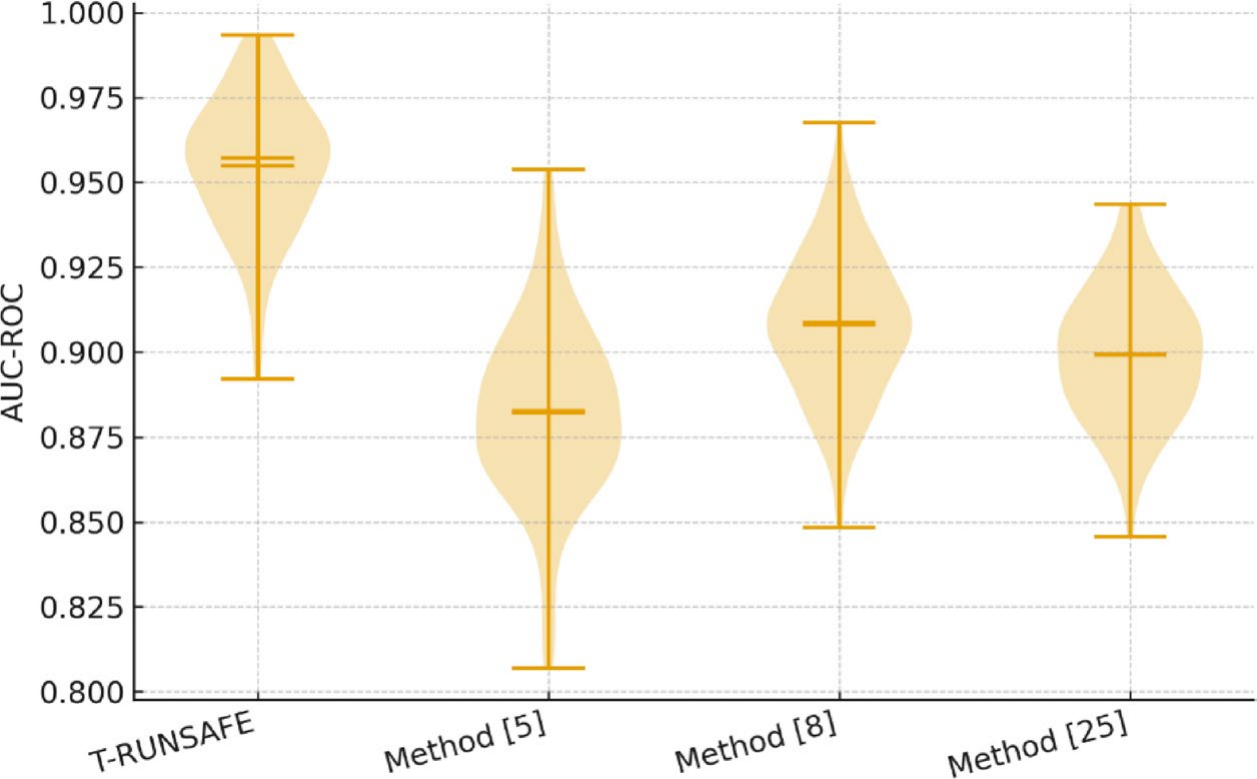
Metric distribution by methods and dataset via Violin plot.

Figure 13 illustrates a violin plot of key performance measures for various datasets and approaches. A violin plot demonstrates data point distribution and density across approaches and datasets using box plots and kernel density plots. Figure 13 compares the performance of multiple methods across two different datasets. The violin plot shows AUC, localization accuracy, and other performance parameters for each approach, with each “violin” representing the range and density of findings. The median value of each method’s performance can be derived from the thick line in the centre of the violin plot. This allows for direct comparisons of the central tendency between methods to show the method consistently achieves higher or lower performance. A plot width indicates the density of the results at that metric value. Performance is more consistent with higher data point concentrations in larger plots. In contrast, narrower sections perform unevenly, suggesting results variability. The violin plot also highlights any outliers, which are represented as individual points outside the main distribution. These outliers could indicate methods that perform exceptionally well or poorly in certain cases. The distribution may reflect trends like one method outperforming others across both datasets and significant variation depending on the dataset.

## Conclusion, limitations & future scopes

This study introduced T-RUNSAFE, a unified multi-modal machine learning framework for predictive thermal runaway risk assessment in high-energy lithium-ion batteries. The framework uniquely integrates spatiotemporal attention (ST-FORMER), cross-modal variational encoding (FUSE-GEN), graph-based degradation modeling (DEGRA-GNN), counterfactual interpretability (CAUS-RUN), and reinforcement learning-driven sensor adaptation (SENSOR-RL), thereby offering a balanced combination of predictive accuracy, interpretability, and real-time operational efficiency. Collectively, these capabilities establish T-RUNSAFE as an accurate, interpretable, and energy-efficient early-warning framework for next-generation battery safety management. The key conclusions of the study are.

- T-RUNSAFE achieved a maximum AUC-ROC of 0.965 for early runaway prediction on NASA PCoE and CALCE thermal abuse datasets.
- Identified early degradation with 93.5% accuracy, reducing missed pre-runaway cases by 28.3%.
- Attained high predictive reliability with a causal fidelity score of 0.894.
- Reduced sensor energy consumption by 37% through dynamic sensor throttling.
- Enhanced model robustness under partial sensor failure or noisy input conditions.
- Establishes T-RUNSAFE as an effective early warning framework for lithium-ion battery safety monitoring.

### Limitations

The T-RUNSAFE framework, while demonstrating high performance, exhibits several limitations. Its reliance on high-resolution thermal and acoustic emission (AE) sensors poses challenges for low-cost applications, as these sensors can be both expensive and complex to implement. The counterfactuals produced by CAUS-RUN are computationally intensive, which may impede real-time deployment. Furthermore, the model’s ability to generalize across different battery chemistries and cell architectures remains limited. It has also not been validated under adverse operating conditions or highly non-stationary scenarios. In addition, the lack of publicly available, spatially resolved ground-truth data on internal degradation restricts the benchmarking of DEGRA-GNN’s spatial predictions against absolute accuracy. Addressing these challenges in future work would enhance the robustness and practicality of the proposed system for real-world deployment.

### Future scopes

T-RUNSAFE is a thermal runaway risk assessment framework that can be enhanced through the integration of the ST-FORMER and DEGRA-GNN modules within physics-informed neural networks (PINNs). Future work should systematically benchmark lab-scale experimental results against real-world drive-cycle data, while also incorporating multi-modal datasets such as electrochemical impedance spectroscopy (EIS) and X-ray computed tomography. In addition, reinforcement learning modules should be transitioned from offline training to online adaptive control. Federated learning approaches are recommended to mitigate privacy and scalability constraints in grid-scale storage and fleet electric vehicle deployments. Furthermore, advances in neuromorphic computing and edge AI accelerators present an opportunity to embed T-RUNSAFE directly into Battery Management Systems (BMS), enabling real-time, onboard, and intelligent safety management.

## Data Availability

The data that support the findings of this study are available within this manuscript.

## Abbreviation

AE: Acoustic Emission
AI: Artificial Intelligence
AUC: Area Under Curve
BMS: Battery Management System
CALCE: Center for Advanced Life Cycle Engineering
CAUS-RUN: Counterfactual Thermal Runaway Attribution
CT: Computed Tomography
CNN: Convolutional Neural Network
DEGRA-GNN: Degradation Graph Neural Network
DFD: Data Flow Diagram
EIS: Electrochemical Impedance Spectroscopy
EV: Electric Vehicle
FPS: Frames Per Second
FFT: Fast Fourier Transform
FUSE-GEN: Fusion Generative Network
GAE: Generalized Advantage Estimation
GAT: Graph Attention Network
GNN: Graph Neural Network
HRR: Heat Release Rate
HR: Hazard Ratio
KL: Kullback–Leibler (divergence)
LIB: Lithium-Ion Battery
LFP: Lithium Iron Phosphate
LNâ: Liquid Nitrogen
LSTM: Long Short-Term Memory
ML: Machine Learning
MSE: Mean Squared Error
NCA: Nickel Cobalt Aluminum
NMC: Nickel Manganese Cobalt Oxide
PCM: Phase Change Material
PCoE: Prognostics Center of Excellence
PINN: Physics-Informed Neural Network
PPO: Proximal Policy Optimization
RL: Reinforcement Learning
ROC: Receiver Operating Characteristic
SA: Safety Assessment
SAW: Surface Acoustic Wave
SCM: Structural Causal Model
SENSOR-RL: Sensor Reinforcement Learning
SOC: State of Charge
SoH: State of Health
TR: Thermal Runaway
VAE: Variational Autoencoder
ViT: Vision Transformer
